# Democratizing wildfire strategies. Do you realize what it means? Insights from a participatory process in the Montseny region (Catalonia, Spain)

**DOI:** 10.1371/journal.pone.0204806

**Published:** 2018-10-16

**Authors:** Iago Otero, Marc Castellnou, Itziar González, Etel Arilla, Llorenç Castell, Jordi Castellví, Francesc Sánchez, Jonas Ø. Nielsen

**Affiliations:** 1 Integrative Research Institute on Transformations of Human-Environment Systems, Humboldt-Universität zu Berlin, Berlin, Germany; 2 Institute of Environmental Science and Technology, Universitat Autònoma de Barcelona, Cerdanyola del Vallès, Spain; 3 Grup de Reforç d’Actuacions Forestals (GRAF), Fire Department, Department of Home Affairs, Catalan Regional Government, Cerdanyola del Vallès, Spain; 4 Institut Cartogràfic de la Revolta, Barcelona, Spain; 5 Geography Department, Humboldt-Universität zu Berlin, Berlin, Germany; Texas A&M University, UNITED STATES

## Abstract

Participatory planning networks made of government agencies, stakeholders, citizens and scientists are receiving attention as a potential pathway to build resilient landscapes in the face of increased wildfire impacts due to suppression policies and land-use and climate changes. A key challenge for these networks lies in incorporating local knowledge and social values about landscape into operational wildfire management strategies. As large wildfires overcome the suppression capacity of the fire departments, such strategies entail difficult decisions about intervention priorities among different regions, values and socioeconomic interests. Therefore there is increasing interest in developing tools that facilitate decision-making during emergencies. In this paper we present a method to democratize wildfire strategies by incorporating social values about landscape in both suppression and prevention planning. We do so by reporting and critically reflecting on the experience from a pilot participatory process conducted in a region of Catalonia (Spain). There, we built a network of researchers, practitioners and citizens across spatial and governance scales. We combined knowledge on expected wildfires, landscape co-valuation by relevant actors, and citizen participation sessions to design a wildfire strategy that minimized the loss of social values. Drawing on insights from political ecology and transformation science, we discuss what the attempt to democratize wildfire strategies entails in terms of power relationships and potential for social-ecological transformation. Based on our experience, we suggest a trade-off between current wildfire risk levels and democratic management in the fire-prone regions of many western countries. In turn, the political negotiation about the landscape effects of wildfire expert knowledge is shown as a potential transformation pathway towards lower risk landscapes that can re-define agency over landscape and foster community re-learning on fire. We conclude that democratizing wildfire strategies ultimately entails co-shaping the landscapes and societies of the future.

## 1. Introduction

As climate change, land-use changes and suppression policies exacerbate the wildfire problem in many western countries, concern emerges on how to coexist with this perturbation [1]. Among the different options debated in the literature, collaborative or participatory planning networks made of public agencies, stakeholders and citizens are receiving attention as a potential tool to build resilience to wildfires [2–7]. Related to this endeavour is the notion of community-based fire management, where local communities plan their own wildfire regimes through partnerships with government agencies, NGOs and the private sector [8]. The development of these networks is driven both by a normative motivation to democratize wildfire management [9] and by the recognition that the complexity of the wildfire problem requires new governance arrangements operating at multiple scales [2,10,11]. This is in line with studies on adaptive governance showing that developing the right links between institutions across multiple organizational levels is crucial to build social-ecological resilience [12–15].

A key challenge for participatory wildfire planning networks lies in incorporating landscape values, local knowledge, and social perceptions of risk into operational decision making systems [9,16–18]. There is a large range of values that might be affected by wildfire events and management [19]. These heterogeneous values are embedded in often conflicting visions and policies of wildfire risk reduction [20]. Moreover, in many western countries local or traditional fire knowledge systems vanished with industrialization [21], and nowadays local communities have very limited experience in fire management as compared to expert agencies [1]. Which and whose values and knowledge are included in participatory wildfire planning networks are crucial questions when trying to build resilient landscapes. New scientific, technical and deliberative methodologies are therefore required, and our paper addresses this need.

Recently some practitioners have stressed the need to open the wildfire strategies of the fire departments up to democratic decision-making. ‘Strategy’ refers to the set of responses applied by the fire department to reduce the uncertainty created by a wildfire, involving a prioritization of the interventions to achieve concrete objectives such as, for instance, confining the wildfire within 150 ha [22,23]. Developing a strategy requires considering the wildfire potential, its suppression opportunities, and the people, land-uses, and properties at risk, as well as making decisions about the wildfire impacts on these factors [22]. As large wildfires increasingly overcome the suppression capacity of the fire departments [24–26], strategies entail difficult decisions about intervention priorities among different areas, landscape values and socioeconomic interests. Therefore there is increasing interest in developing tools to include the values and interests at stake into operational wildfire strategies, in order to facilitate decision-making processes during emergencies [27]. While ‘strategy’ mostly refers to the management of wildfires when they occur, it can be planned in advance by anticipating the wildfire patterns of a certain region and planning preventive landscape interventions. Thus, in this paper ‘strategy’ encompasses both prevention and suppression practices.

In La Jonquera large wildfire (Catalonia, Spain, 2012), one of us (M.C., GRAF´s analyst) opened up the strategy of the Fire Department by reaching an agreement on intervention priorities with politicians, firefighters and stakeholders of the affected landscapes during an improvised meeting. Since then similar approaches were used during all significant wildfires occurring in Catalonia. Based on that first experience, an attempt was made to plan participatory strategies with stakeholders without the pressure of the emergency, in Eastern Mourns (Northern Ireland, UK). In theory, the knowledge about wildfire patterns of a given region can provide scenarios to decision making, and planning the strategies in advance has the potential to reduce uncertainty during emergencies.

However, operationalizing this call is far from easy. As we show in this paper, the attempt to democratize wildfire strategies not only adds complexity to the challenge of developing participatory planning networks which are sensitive to social values about landscape, but it has also dramatic implications in terms of power relationships, agency over landscape and the potential for social-ecological transformation in fire-prone regions. We present a method to democratically plan wildfire strategies by reporting and critically reflecting on the experience from a pilot participatory process conducted in the Montseny region (Catalonia, Spain). Drawing on insights from political ecology [28–30], transformation science [31–34] and action-research [35,36] we argue that democratizing wildfire strategies ultimately entails co-shaping the societies and landscapes of the future.

The paper is structured as follows. Section 2 describes the study region. Section 3 explains the participatory process for the democratization of wildfire strategies which combines knowledge on expected wildfires, landscape co-valuation by relevant actors, and citizen participation sessions. In section 4 we critically reflect on the process based on an analysis of I.O.’s project diary and other documents such as minutes of meetings and presentations. Section 5 discusses the implications of our experience and section 6 concludes.

## 2. Context: The Montseny region

### 2.1. General features

**Fig 1:**
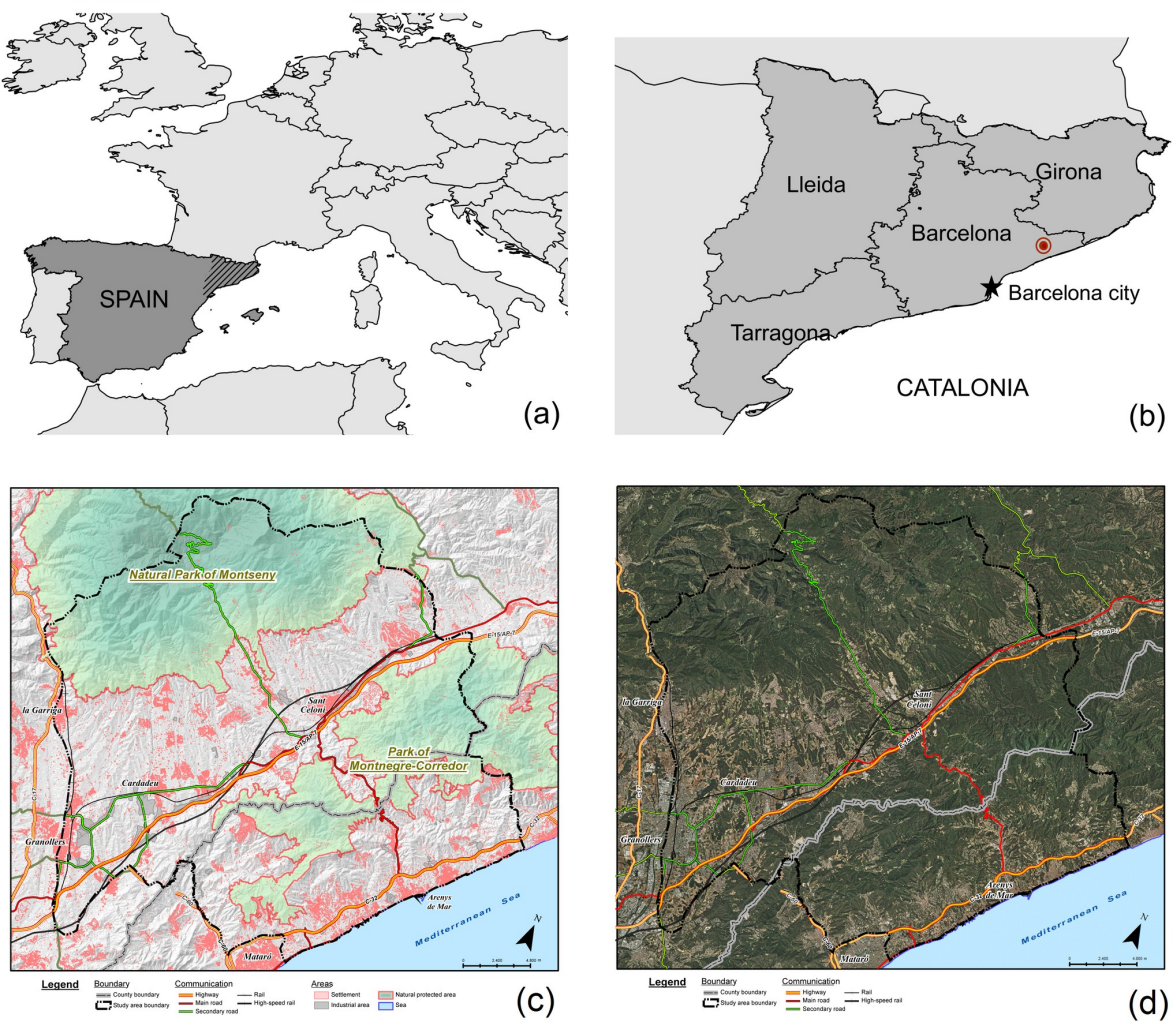
Presentation of the study region. a) Catalonia within Spain and Europe. b) Location of the Montseny region within the Barcelona province, in Catalonia. c) Main territorial characteristics of the Montseny region. d) Orthophoto of the Montseny region (2015). Settlement, industrial activities and strategic transport infrastructures in extensively forested mountain ranges make the region highly vulnerable to large wildfires. Source: own elaboration with data from Institut Cartogràfic i Geològic de Catalunya and Instituto Geográfico Nacional. Note: “Cardadeu” is misspelled. The correct spelling is “Cardedeu”.

A region of 60,596 ha was selected in the Barcelona metropolitan region, belonging to the autonomous region of Catalonia in north-eastern Spain (Fig 1A and 1B). The region includes the pre-littoral ranges of Montseny–reaching ca. 1700 m a.s.l.–and the littoral ranges of Montnegre-Corredor–reaching ca. 760 m a.s.l. (Fig 1C). Between the two ranges lay the large plain of Granollers, drained by the Mogent river, and the narrow plain of Sant Celoni, along which the Tordera river flows. The highway AP-7 and the high-speed train–connecting the Spanish and French coasts–pass through these plains, which host a large share of the region’s population as well as most of its industrial activities (Fig 1C and 1D). The coastal strip of the region has a highway and hosts several touristic towns. Settlement is structured in towns or cities, sprawled residential developments, and isolated farmhouses. The city of Granollers with ca. 60,000 inhabitants, and several cities between 15,000 and 20,000 inhabitants such as Cardedeu, Sant Celoni or Arenys de Mar are placed in the region. The inland municipalities belong to the Vallès Oriental county and the coastal ones belong to the Maresme county.

The region has gone through a dramatic rural-to-urban socioeconomic transformation during the 20^th^ century. Cropland and pastureland has shrunk while forests, urban and industrial areas have expanded. This has led to substantive changes in landscape ecology and biodiversity [37] and to a very vulnerable landscape concerning wildfires [38]. Both mountain ranges are partly protected by natural parks, the Montseny one being a UNESCO Biosphere Reserve. All these changes have gone hand in hand with an erosion of local fire knowledge, especially in the plateaus of Montseny where the traditional fires performed by shepherds for pasture regeneration were banned by the Natural Park in the early 1980s [39]. Forestland ownership in our study region is mostly private, even if some estates within the natural parks are owned by a public agency (the Barcelona Province Authority, in charge of managing the natural parks).

### 2.2. Wildfire pattern

**Fig 2:**
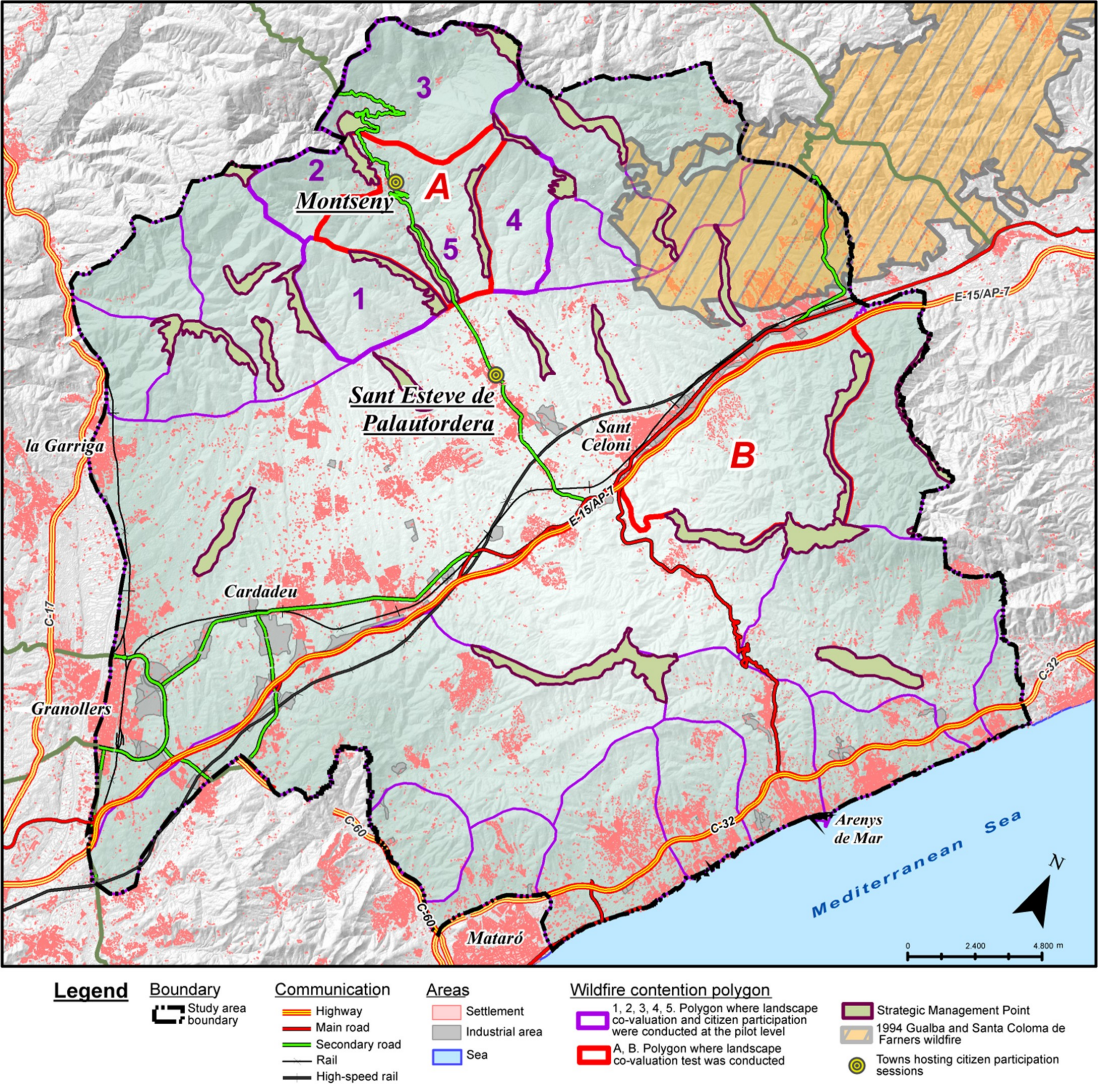
Wildfire pattern and participatory dynamics in the Montseny region. The region was divided in wildfire contention polygons (purple lines) and suggested strategic management points (light green areas). A method for landscape co-valuation was tested in polygons A and B (red lines). Landscape co-valuation and citizen participation sessions were conducted at the pilot level in polygons 1 to 5. The towns hosting citizen participation sessions (Montseny and Sant Esteve de Palautordera) are indicated. The 1994 large wildfire’s perimeter is likewise shown. Source: own elaboration with data from Institut Cartogràfic i Geològic de Catalunya, Instituto Geográfico Nacional and Catalan Fire Department. Note: “Cardadeu” is misspelled. The correct spelling is “Cardedeu”.

The wildfire pattern of the region is mostly composed by convection dominated wildfires with wind, but it also includes topographic wildfires [40,41]. In convection dominated wildfires, the wildfire’s convective column transmits heat and throws spots to the surroundings thereby accelerating combustion. Wind increases spotting distance, creating new ignitions outside the convective column’s influence zone and further accelerating wildfire spread. High fuel availability in the forests, stemming from historical landscape changes, determines high wildfire intensity. With these conditions, the wildfire quickly overcomes the Fire Department’s suppression capacity. The largest historical wildfire in the region responded to this pattern. This wildfire resulted from the union of two wildfires that started in Gualba and Santa Coloma de Farners on the 10^th^ August 1994. They united two days later, affecting about 11,000 hectares (Fig 2). In that event, west winds, local winds, accumulated drought, very high temperatures, relative humidity below 30% and fuel continuity combined to produce an out-of-control wildfire [42]. Topographic wildfires are instead driven by local topographic winds and follow the slopes with the steepest gradient and highest insolation; they also include wildfires driven by the turn of the sea breezes [41]. In worst case scenarios, in our study region the Fire Department expects simultaneous large wildfires with virulent behaviour causing civil emergencies, as they threaten residential areas and key economic activities and transport routes (Fig 1C and 1D).

### 2.3. Wildfire governance system

The wildfire governance system in the region is composed of several institutions operating at different scales, whose interactions drive management outcomes on the ground. The town councils implement municipal wildfire prevention schemes with the support of the Technical Office of Municipal Wildfire Prevention of the Barcelona Province Authority (TOMWP) and in collaboration with the forest defence associations. The latter are heterogeneous municipal or multi-municipal groups of forest landowners, town councils and volunteers working with prevention and auxiliary extinction. Forest defence associations federate at the county level. During a wildfire, they are under the command of the Fire Department, which deals with the suppression operations and the protection of people.

The Fire Department belongs to the Department of Home Affairs of the Catalan regional government and operates at the Catalan scale. It is organized in seven emergency regions. GRAF is the Fire Department’s wildfire specialists group, and has a technician in each emergency region. In the Northern Metropolitan Emergency Region where our study region belongs, GRAF’s technician plans low fuel strips in collaboration with the TOMWP, forest landowners and the Forest Property Centre. Forest landowners form associations at the scale of mountain range (one in Montseny and one in Montnegre-Corredor). These associations defend the interests of private forest owners before public agencies. The one in Montnegre-Corredor develops forest planning for wildfire prevention with the support of the TOMWP. The Forest Property Centre is a public agency enhancing forest planning in private forests across Catalonia through the approval of estate and municipal schemes, often with wildfire prevention as a criterion. The natural parks are managed by the Barcelona Province Authority and have their own wildfire prevention schemes. These mostly consist in the management of forest tracks and water infrastructures. Finally, the county offices of the Barcelona Territorial Services of the Catalan Department of Agriculture implement general directives from the Department’s Services of Wildfire Prevention and Forest Management.

## 3. The participatory process: A method for the democratization of wildfire strategies

### 3.1. Motivations

The method for the democratization of wildfire strategies evolved out of the synergies between the motivations of the core team (Table 1). These motivations ranged from a theoretical interest in understanding the social-ecological reconfigurations needed to coexist with wildfire to a political commitment towards better public services, more democratic politics and social-ecological transformation. The participatory process was specifically part of the postdoctoral project “The political ecology of wildfires” developed by I.O. at IRI THESys [1,43]. It was conducted in the region where he used to live during the project’s fieldwork, as an action-research project towards alternative, less flammable landscapes [36,44].

**Table 1:** Composition and motivations of the core team of the participatory process. The roles performed by each institution are shown in S1 Table. Source: own elaboration.

Person	Institution	Motivations
Iago Otero	Integrative Research Institute on Transformations of Human-Environment Systems (IRI THESys), Humboldt University of Berlin (Germany)	1. Understand how social groups reconfigure their relations with the environment as they learn to coexist with wildfire. 2. Contribute to initiate radical transformations towards alternative landscapes that are unable to burn at high intensity. 3. Link wildfire prevention to alternative forest and land management practices embedded in regional networks of production and consumption.
Marc Castellnou	Support Group for Forest Interventions (GRAF), Fire Department, Department of Home Affairs, Catalan regional government (Spain)	1. Democratize the strategies of the Fire Department to prevent, suppress and manage wildfires, moving from technical to social strategies. 2. Include the conflicting social values about landscape in the design of wildfire strategies, moving from the minimization of area burnt to the minimization of value loss. 3. Increase the efficiency of the public service in the management of wildfire emergencies by introducing the common good in front of private interests.
Itziar González	Association Cartographic Institute of Revolt (ICR), Barcelona (Spain)	1. Overcome barriers for inter-agency and citizen-agency cooperation and trust, including non-transparency, corruption, and non-accountability. 2. Empower citizens in the stewardship of common resources through participatory learning processes. 3. Map emerging practices and networks of democratic politics.

As the head of GRAF, M.C. is in charge of developing the strategies followed by the Fire Department during wildfires across Catalonia. In talks to wildfire experts and practitioners he had previously stressed the need to democratize those strategies; something he thought should be done by including the often conflicting social values about landscape in their design. He aimed at doing so in this participatory process. The latter ambition was intended to strengthen the quality and accountability of the public service, in the face of financial cuts and potential privatization after the economic and debt crisis. Indeed, in Catalonia and Spain, the latter intermingled with a crisis of democratic institutions, with corruption eroding the trust of citizens, who mobilized in new practices of “real democracy” [45]. In this context, the main motivation of I.G. and her association was to empower citizens in the stewardship of common resources through renewed mechanisms of accountability of the public administration.

**Fig 3:**
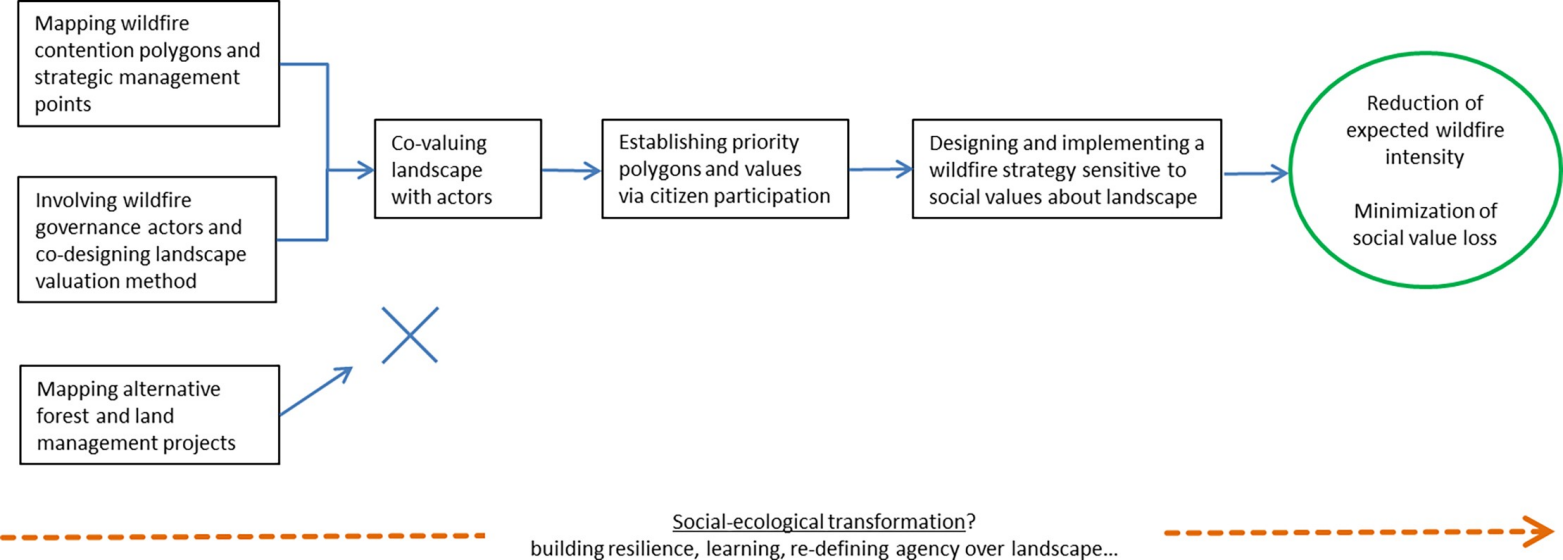
Participatory process for the democratization of wildfire strategies. Rectangles show the main steps, and blue arrows show the (dis)connection between them. The green oval outlines the potential concrete outcomes of the process. Underlying the participatory process, there is a potential transformative pathway towards a new social-ecological setup. See text for details. Source: own elaboration.

In the next sub-sections we summarize the main steps of the participatory process (see also Fig 3). A detailed timeline including the main tasks and the responsible institutions can be found in the supporting information (S1 Table). In what follows, ‘facilitators’ refers to IRI THESys and ICR (Table 1).

### 3.2. Mapping wildfire contention polygons and strategic management points based on the expected wildfire pattern

We started by drawing wildfire contention polygons in a Geographic Information System (GIS) (Figs 2 and 3). These were based in GRAF’s knowledge of the wildfire pattern, stemming from a study of past wildfires [40]. In our study region, expected wildfires include topographic wildfires and convection dominated wildfires with wind [41]. Wildfire contention polygons typically follow crest lines and other topographic elements where wildfires change behaviour. They are used by GRAF analysts to assess wildfires’ spreading pattern and potential size according to meteorological, topographic and fuel conditions, as well as to the effects of the implemented strategies. Essentially, they are areas within which a wildfire might be contained thus avoiding its propagation to neighbouring polygons.

Wildfire contention polygons were used as the basis of a participatory landscape valuation that would inform about the social priorities–i.e. which polygons were most valued and why–in order to translate them into concrete strategies. Strategies include strategic management points (SMP), which are wildfire friendly landscape structures that facilitate suppression and limit wildfire size (Fig 2). They are normally planned along crest lines and in valley bottoms, thus fragmenting the landscape and making fire spread more difficult.

### 3.3. Involving the actors of the wildfire governance system and co-designing the landscape valuation method

We identified the wildfire governance system, i.e. actors with competencies in wildfire prevention, wildfire extinction and forest management operating in our study region (Tables 2 and 3; Fig 3). This identification was done by drawing on the first author experience in wildfire governance projects across Catalonia. Their main scale of intervention ranged from Catalan to municipal. Most of the actors operated at intermediate scales, defined by administrative (province, county) or landscape (mountain ranges) boundaries.

**Table 2:** Representation of the wildfire governance system of our study region, including the main actors and their scale of intervention. This representation was done for the purposes of the present participatory process, and it necessarily involved the inclusion of some actors and the exclusion of others (see Section 4.2). Source: own elaboration.

Scale of intervention	Actor(s)
Autonomous region of Catalonia	GRAF FPC
Barcelona province	TOMWP
Northern Barcelona Metropolitan Region	NMER
Montseny mountain ranges	MAFL NPM
Montnegre-Corredor mountain ranges	MCAFL PMC
County of Vallès Oriental	VOCO FFDAVO
County of Maresme	MCO FFDAM
Multiple municipalities	FDAs
Municipality	TOMWP Town Councils FDAs

GRAF: Support Group for Forest Interventions, Fire Department, Catalan Department of Home Affairs.

FPC: Forest Property Centre, Catalan Department of Agriculture.

TOMWP: Technical Office of Municipal Wildfire Prevention, Barcelona Province Authority.

NMER: Northern Metropolitan Emergency Region, Fire Department, Catalan Department of Home Affairs.

MAFL: Montseny Association of Forest Landowners.

NPM: Natural Park of Montseny, Barcelona Province Authority.

MCAFL: Montnegre-Corredor Association of Forest Landowners.

PMC: Park of Montnegre-Corredor, Barcelona Province Authority.

VOCO: Vallès Oriental County Office, Barcelona Territorial Service, Catalan Department of Agriculture.

FFDAVO: Federation of Forest Defence Associations Vallès Oriental County.

MCO: Maresme County Office, Barcelona Territorial Service, Catalan Department of Agriculture.

FFDAM: Federation of Forest Defence Associations Maresme County.

FDAs: Forest Defence Associations.

**Table 3:** Actors participating in the process. In meetings, each actor was characteristically represented by 1–2 persons. Besides the representative/s, other members of the institutions were regularly informed of the process’ developments. Group 3 actors included the town mayor and/or some town councillors. In some cases the mayor represented both the town council and the local forest defence association. Source: own elaboration.

Actor	Scale of intervention	Reasons for inclusion in the process
*GROUP 1*: *Actors of the wildfire governance system operating at scales comprised between the autonomous region of Catalonia and the county*		
Support Group for Forest Interventions (GRAF), Fire Department, Catalan Department of Home Affairs	Autonomous region of Catalonia	Before a wildfire, it plans prevention works and forecasts risk. During the wildfire, it develops the strategy as decision support to the emergency’s head.
Forest Property Centre, Catalan Department of Agriculture	Autonomous region of Catalonia	Public administration that enhances forest planning and management in private forests by means of schemes at the estate and municipal levels. Their guidelines for forest management integrate the prevention of large wildfires as a key objective.
Technical Office of Municipal Wildfire Prevention, Barcelona Province Authority	Municipalities of the Barcelona province	It drafts wildfire prevention schemes for municipalities, including forest tracks, water infrastructures and the protection of residential areas. These schemes are developed in cooperation with the town councils and the forest defence associations. It also develops forest planning for wildfire prevention in private estates in collaboration with the Montnegre-Corredor Association of Forest Landowners.
Northern Metropolitan Emergency Region, Fire Department, Catalan Department of Home Affairs	Northern Barcelona Metropolitan Region	It manages the resources of the region’s fire stations, coordinates prevention tasks, plans activities related to risk monitoring, and commands the operations during wildfire suppression.
Montseny Association of Forest Landowners	Montseny mountain ranges	It groups together Montseny forest landowners. It aims at uniting efforts, looking for new agricultural and forest management opportunities, and disseminating the socio-environmental benefits of sustainable resource management in Montseny.
Natural Park of Montseny, Barcelona Province Authority	Montseny mountain ranges	It manages a significant part of the study area with the aim of making compatible the conservation of natural, landscape and cultural values with socioeconomic development and public use. It manages forest tracks as part of its own wildfire prevention scheme. The Natural Park is a UNESCO Biosphere Reserve.
Montnegre-Corredor Association of Forest Landowners	Montnegre-Corredor mountain ranges	It acts as a representative of Montnegre-Corredor forest landowners before governmental agencies. It aims at enhancing the profitability of forestry and at recovering human population in the mountain farmhouses, abandoned after rural outmigration.
Park of Montnegre-Corredor, Barcelona Province Authority	Montnegre-Corredor mountain ranges	It manages a significant part of the study area with the aim of making compatible the conservation of landscape values with socioeconomic development. It manages forest tracks and water infrastructures as part of its own wildfire prevention scheme.
Vallès Oriental County Office and Maresme County Office, Barcelona Territorial Service, Catalan Department of Agriculture	Counties of Vallès Oriental and Maresme	They implement general directives from the Department´s Wildfire Prevention Service and Forest Management Service. They authorize forestry works in those estates that do not have a planning scheme approved by the Forest Property Centre.
Federation of Forest Defence Associations Vallès Oriental County	County of Vallès Oriental	It coordinates the activities of the county’s forest defence associations (see Group 3, this table).
*GROUP 2*: *Actors with a regional focus providing complementary information on landscape values*		
Coordination Group for Montseny Defence	Montseny and Montnegre-Corredor mountain ranges	Environmentalist platform leading the social movement for the protection of Montseny since the 1980s. It provided polygon-specific information on landscape ecological and cultural values, mainly on the need to recover human population in the mountain after decades of land abandonment.
Museum of Natural Sciences in Granollers	County of Vallès Oriental and mountain ranges of Montseny and Montnegre-Corredor	It monitors biodiversity in the study region. It provided polygon-specific information on the state of small mammals, bats, and butterflies, including habitats and species of special conservation interest.
Department of Food and Animal Science, Autonomous University of Barcelona	Multiple	It develops research on plant ecological responses to grazing and on silvopastoral management alternatives. It provided polygon-specific information on vegetation diversity and proposals to recover open habitats linked to grazing activities in mountains.
Montseny Ethnology Museum in Arbúcies	Montseny mountain ranges	Centre of exhibition, conservation, dissemination and research on Montseny´s cultural heritage. It provided polygon-specific information on archaeological and architectonic elements, as well as on ancient trees.
Centre for Ecological Research and Forestry Applications, and University of Barcelona	Multiple	It develops research on ecosystem structure and functioning; carbon and water balances; and ecophysiological responses of forests to climate change, droughts, wildfires and management. It provided general guidelines on how to manage forests to adapt to climate change, droughts and wildfires.
BeWater Project, Centre for Ecological Research and Forestry Applications	Tordera river catchment	Project promoting science-society collaboration for sustainable water management and adaptation to the impacts of global change in the Mediterranean. The Tordera is one of the river basins studied in the project. It provided general information on the relationship between forest cover and water resources, as well as silvopasture management alternatives.
Forest Museum	Mountain ranges of Montseny and Montnegre-Corredor	Project to create a forest museum in the town of Sant Celoni around the social-environmental heritage of forests. It did not provide information.
*GROUP 3*: *Actors of the wildfire governance system operating in the 5 selected polygons for pilot valuation (municipal scale)*		
Town Council of Sant Esteve de Palautordera	Municipality of Sant Esteve de Palautordera	Municipalities included in the 5 pilot polygons. Town councils develop municipal wildfire prevention schemes with the support of the Technical Office of Municipal Wildfire Prevention of the Barcelona Province Authority and in collaboration with the forest defence associations. Schemes include: maintenance of forest tracks and water infrastructures, protection of residential areas, surveillance, and emergency planning.
Town Council of Montseny	Municipality of Montseny	
Town Council of Sant Pere de Vilamajor	Municipality of Sant Pere de Vilamajor	
Town Council of Fogars de Montclús	Municipality of Fogars de Montclús	
Forest Defence Association of Sant Esteve de Palautordera	Municipality of Sant Esteve de Palautordera	Forest defence associations covering the municipalities included in the 5 pilot polygons. Forest defence associations are made up of forest landowners, town councils and volunteers. They can cover one or more municipalities. They are engaged in prevention activities, which they develop in collaboration with the town councils, as well as in auxiliary extinction activities.
Forest Defence Association of Sant Pere de Vilamajor, Sant Antoni de Vilamajor and Cardedeu	Municipalities of Sant Pere de Vilamajor, Sant Antoni de Vilamajor and Cardedeu	
Forest Defence Association of Montseny-Migjorn	Municipalities of Montseny, Fogars de Montclús and Campins	

A first group of actors from the wildfire governance system was selected including those operating at scales comprised between the autonomous region of Catalonia and the county. Group 1 actors included public agencies, associations of forest landowners, and public-private entities such as a federation of forest defence associations (Table 3). Between March and September 2015 individual meetings were held with these actors to present the project and involve them. A first joint meeting occurred in October 2015. In that meeting, the goals of the project and the methods of landscape valuation were discussed. As a follow-up of the meeting, we did a landscape valuation test in 2 polygons (1 in Montnegre-Corredor mountain range and 1 in Montseny mountain range; polygons A and B in Fig 2). For this, we asked actors to provide a text describing their interests and/or law-mandated competencies, including landscape values, wildfire and forest management tasks, challenges and proposals. They were asked to provide information in a way that would help citizens appreciate the specific value of the polygons during the participatory sessions that would be organized in the future.

**Fig 4:**
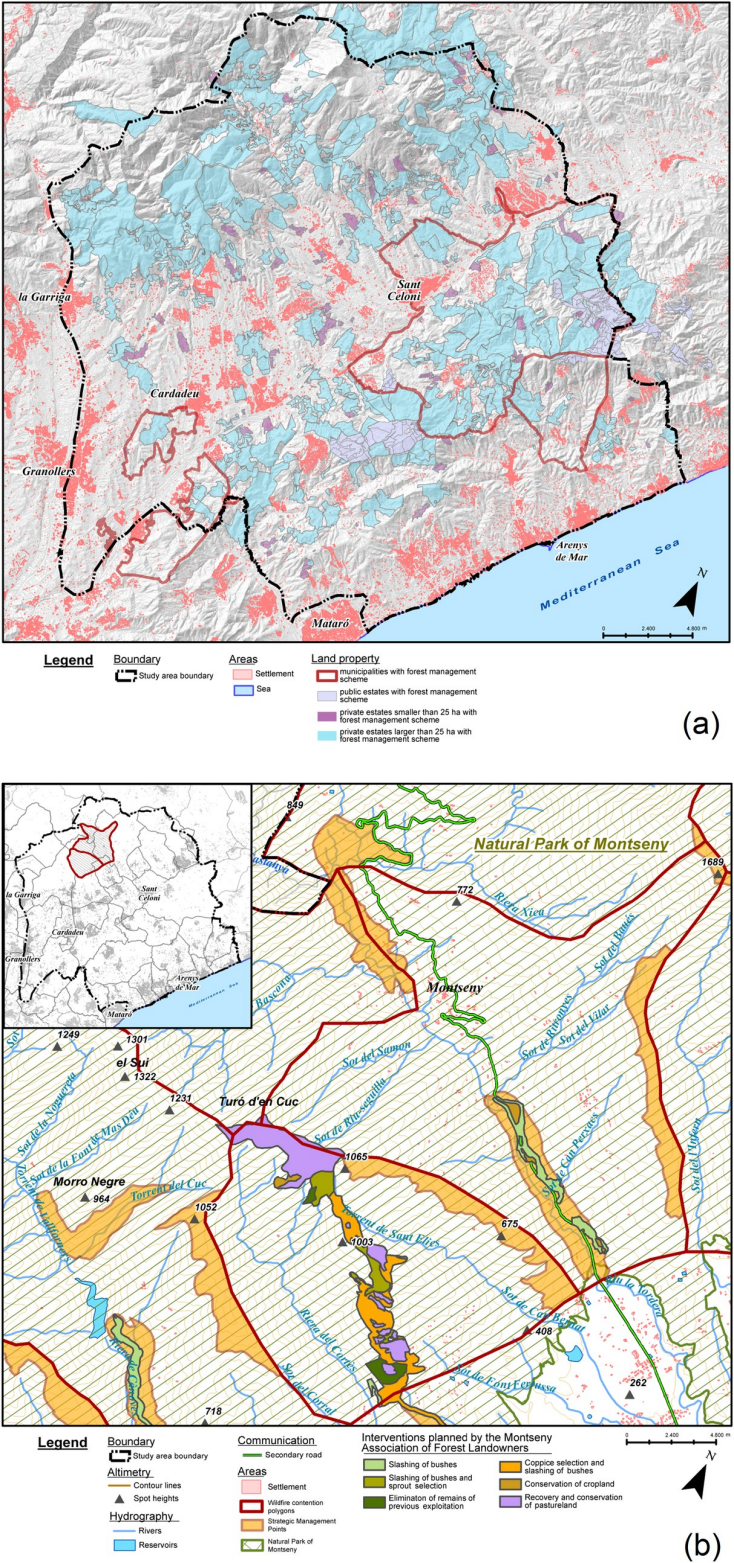
Common GIS. Displaying and overlapping the GIS layers provided by the actors supported the landscape co-valuation exercise and was key to build a legitimate participatory process. Here we provide two examples. a) Forest management schemes in force, provided by the Forest Property Centre. The extent of land under planned forest management served as an indication of the potential to develop joint efforts for wildfire prevention, landscape management and enhancement of regional economic activities. Overlapping this layer with the one on settlements made clear a challenge for wildfire risk reduction: the integration of forest and urban planning, currently under disconnected agencies. b) Strategic management points planned by the Fire Department and landscape management planned by the Montseny Association of Forest Landowners. The common GIS allowed identifying areas of convergence and complementarity between public wildfire prevention criteria and private landscape management interests, revealing potential synergies between actors. Source: own elaboration with data from Institut Cartogràfic i Geològic de Catalunya, Instituto Geográfico Nacional, Forest Property Centre, Montseny Association of Forest Landowners and Catalan Fire Department. Note: “Cardadeu” is misspelled. The correct spelling is “Cardedeu”.

We did a synthesis of the contributions and discussed it with the actors during a second meeting held in January 2016. We coded the synthesis of polygon A and created six categories of landscape values (Biodiversity and cultural heritage, Current socioeconomic activities, Cooperative fabric, Elements in need of special protection during wildfire; Potential for social and ecological economy; and Other values). Based on these categories, a sheet was designed to gather polygon-specific input on landscape values from actors (Table 4). The landscape valuation design was supported by the creation of a GIS composed of the layers used by the different actors in their wildfire and forest management tasks (Fig 4).

**Table 4:** Landscape valuation sheet administered to group 1 and 2 actors to value the 5 polygons selected for pilot citizen valuation. 1 = less importance, 5 = more importance. Translated from Catalan. Source: own elaboration.

Name of contributing actor: Polygon number: Date:	
A. Biodiversity and cultural heritage (max. 100 words)	Importance: (1–5)
B. Current socioeconomic activities (max. 100 words)	Importance: (1–5)
C. Cooperative fabric (max. 100 words)	Importance: (1–5)
D. Elements in need of special protection during wildfire (max. 100 words)	Importance: (1–5)
E. Potential for social and ecological economy (max. 100 words)	Importance: (1–5)
F. Other values (max. 100 words)	Importance: (1–5)

Explanation of categories: A: Intra- and inter-specific diversity as well as environmental diversity, intricately linked to the structure and dynamics of a cultural landscape including archaeological and architectonic elements, agro-silvo-pastoral management systems and intangible heritage. It can include the state, threats, potential and protection figures of all these values. B: Settlement patterns and current economic activities including forest estates (structure, land-use types, whether they have planning schemes in force, main obstacles to enhance economic activities based on local forest resources). C: Cooperation networks between people, between estates, between forest landowners and governmental agencies or between forest landowners and local and regional economic sectors. They include associations of forest owners, forest defence associations, public-private partnerships for forest planning or commercial networks of regional products. D: Elements especially vulnerable to wildfire, including houses, certain forest uses or key economic activities at the local and regional level. E: Potential for new economic activities and employment based on an enhancive use of local resources (forests, pastures, cropland, water, farmhouses, services, socioeconomic networks) as a source of wellbeing. It also includes intervention proposals to build resilience to wildfire and the explanation of their effects on wildfire behaviour. F: Any value not mentioned in the former categories.

A second group of actors (Group 2, Table 3) was selected that could provide complementary information on diverse landscape values ranging from biodiversity and cultural heritage to land management options. They had mostly a regional focus and included museums, universities and an environmentalist platform. Representatives from these actors were invited to a joint meeting in January 2016 where the project was presented.

### 3.4 Mapping alternative forest and land management projects

A map of alternative land-use projects was started by I.O. and his commune’s housemates, interested in building a network of people with experience in agro-ecological land reclamation practices (Fig 3). These practices were considered to have the potential to influence a shift towards less flammable landscapes through forestry, grazing and agriculture. At the same time, they could contribute to re-frame the wildfire issue as a matter of transformation of the current land-use model, characterized by the abandonment of forest and land management in mountain ranges. This map was expected to converge with the landscape valuation and the GIS developed with the actors. 28 projects were selected 1) working in the region’s forests, croplands and pasturelands, 2) selling or buying products resulting from these, and 3) working towards the revitalization of rural activities and the enhancement of regional networks of production and consumption. An interview guide was designed to gather basic information about the projects and the kind of transformation they wanted to contribute to, as well as about the main challenges and suggested solutions. Four of them were visited and their promoters interviewed, including producers of artisan cheese, organic meat and firewood. However, the mapping was aborted due to fading of initial enthusiasm and lack of human resources.

### 3.5. Co-valuing landscape in a pilot area

Given the complexity of conducting a landscape co-valuation in the entire study region, we selected five contiguous polygons in the Montseny range to conduct a pilot citizen valuation (Fig 2, polygons 1–5; Fig 3). Actors from groups 1 and 2 were given the task of supplying information on landscape values that would be used to inform citizens´ prioritization of polygons. For this, we administered the landscape valuation sheet to them, asking to fill out one sheet per polygon (Table 4). This sheet included qualitative information on each category of landscape values as well as a numerical assessment of their relative importance as compared to the other polygons. Actors were asked to work with those categories relevant to their competency or expertise and leave the rest blank.

In February and March 2016 we gathered and synthesized the inputs from the valuation sheet. We received texts on one or more of the 6 categories from 13 actors. 11 of these actors provided polygon-specific valuations, while two of them provided general information or guidelines. We received numerical assessments (1–5 ranking) from 9 actors. Two actors teamed up to perform the exercise as they belonged to the same agency. We synthesized the texts per category and polygon, while checking the veracity of information and trying to keep the original diversity and nuances. We calculated the average values per category, polygon, and actor group (Table 5). These results were sent back to the actors together with an explanation of the methods used to synthesize the information. The ranking of polygons differed between actors in group 1 (P5>P4>P1>P3>P2) and group 2 (P3>P5>P1>P4>P2).

**Table 5:** Results of the landscape valuation exercise (quantitative part) based on input from group 1 and 2 actors. Lower case letters *a* to *i* refer to actors that provided quantitative assessments (n = 9, note that two actors teamed up to perform this exercise). Capital letters *A* to *F* refer to categories of values. Scores ranged from 1 (less importance) to 5 (more importance). nv: not valued. Source: own elaboration.

	Polygon 1						Polygon 2						Polygon 3						Polygon 4						Polygon 5					
	A	B	C	D	E	F	A	B	C	D	E	F	A	B	C	D	E	F	A	B	C	D	E	F	A	B	C	D	E	F
a	nv	4	4	nv	4	nv	nv	3	3	nv	2	nv	nv	5	4	5	5	nv	nv	5	4	nv	5	nv	nv	5	4	5	5	nv
b	nv	5	5	nv	nv	nv	nv	5	5	nv	nv	nv	nv	5	5	nv	nv	nv	nv	5	5	nv	nv	nv	nv	5	5	nv	nv	nv
c-d	nv	nv	nv	4	1	4	nv	Nv	nv	1	nv	2	nv	nv	nv	3	nv	1	nv	nv	nv	4	nv	3	nv	nv	nv	5	4	5
e	nv	5	5	nv	nv	nv	nv	5	5	nv	nv	nv	nv	5	5	nv	nv	nv	nv	5	5	nv	nv	nv	nv	5	5	nv	nv	nv
f	5	3	nv	5	5	4	5	3	nv	5	5	4	5	5	nv	5	5	5	5	4	nv	3	5	5	4	3	nv	4	5	4
g	3	2	nv	nv	nv	nv	5	4	nv	5	2	nv	5	4	nv	5	2	nv	5	4	nv	nv	2	nv	4	3	nv	nv	nv	nv
h	4	nv	nv	nv	nv	nv	4	Nv	nv	nv	nv	nv	5	nv	nv	nv	nv	nv	3	nv	nv	nv	nv	nv	3	nv	nv	nv	nv	nv
i	4	nv	3	5	nv	4	4	Nv	2	4	3	4	5	nv	3	5	4	4	4	nv	3	4	nv	4	4	nv	nv	4	nv	4
**Category average**	**4.00**	**3.80**	**4.25**	**4.67**	**3.33**	**4.00**	**4.50**	**4.00**	**3.75**	**3.75**	**3.00**	**3.33**	**5.00**	**4.80**	**4.25**	**4.60**	**4.00**	**3.33**	**4.25**	**4.60**	**4.25**	**3.67**	**4.00**	**4.00**	**3.75**	**4.20**	**4.67**	**4.50**	**4.67**	**4.33**
actor group 1 (a-e)	nv	4.67	4.67	4.00	2.50	4.00	nv	4.33	4.33	1.00	2.00	2.00	nv	5.00	4.67	4.00	5.00	1.00	nv	5.00	4.67	4.00	5.00	3.00	nv	5.00	4.67	5.00	4.50	5.00
actor group 2 (f-i)	4.00	2.50	3.00	5.00	5.00	4.00	4.50	3.50	2.00	4.67	3.33	4.00	5.00	4.50	3.00	5.00	3.67	4.50	4.25	4.00	3.00	3.50	3.50	4.50	3.75	3.00	nv	4.00	5.00	4.00
**Polygon average**	**4.01**						**3.72**						**4.33**						**4.13**						**4.35**					
actor group 1 (a-e)	3.97						2.73						3.93						4.33						4.83					
actor group 2 (f-i)	3.92						3.67						4.28						3.79						3.95					

Actors: a) Forest Property Centre, Catalan Department of Agriculture; b) Federation of Forest Defence Associations Vallès Oriental County; c) Support Group for Forest Interventions (GRAF), Fire Department, Catalan Department of Home Affairs; d) Northern Metropolitan Emergency Region, Fire Department, Catalan Department of Home Affairs; e) Technical Office of Municipal Wildfire Prevention, Barcelona Province Authority; f) Coordination Group for Montseny Defence; g) Department of Food and Animal Science, Autonomous University of Barcelona; h) Museum of Natural Sciences in Granollers; i) Montseny Ethnology Museum in Arbúcies.

Categories: A) Biodiversity and cultural heritage; B) Current socioeconomic activities; C) Cooperative fabric; D) Elements in need of special protection during wildfire; E) Potential for social and ecological economy; F) Other values (see description of categories in Table 4).

### 3.6. Preparing participatory exhibitions in the pilot area

In parallel, a third group of actors from the wildfire governance system was selected including those operating at the scale of the 5 polygons chosen for citizen valuation, i.e. municipality and multi-municipality (Table 2). Group 3 actors included the town councils and the local forest defence associations of the municipalities included in those polygons (Table 3). Specific meetings with mayors were held in January and April 2016 to explain the project and co-involve them in the organization of exhibitions for citizen valuation. Next a joint meeting occurred with the mayors, some of the councillors and the heads of the local forest defence associations, where the functioning of the exhibitions was explained in detail.

**Fig 5:**
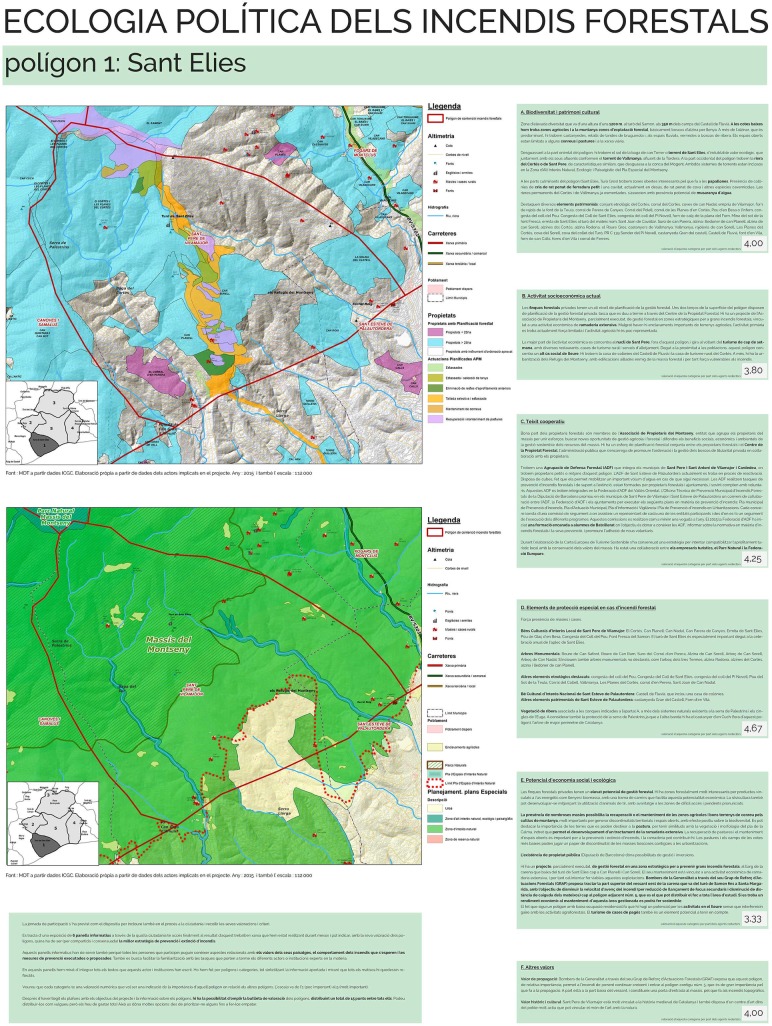
Example of the posters used in the participatory exhibitions to highlight the values found in each polygon (polygon 1 poster). The maps showed the logic of creating a common GIS, where values and land-use conflicts and synergies among actors could be visualized. As an example, in the posters we compared the map of the Montseny Association of Forest Landowners and the Forest Property Centre (upper part) with the zoning of the Montseny Natural Park (lower part; zoning currently annulled). In the right column there was a box for each category of landscape values, with a synthesis of the values found for that category, and the average numerical assessment of their relative importance as compared to the rest of the polygons. Both the text and the numerical assessment came from the input of group 1 and 2 actors. Source: own elaboration based on input from the participatory process. The maps within the poster contain data from Institut Cartogràfic i Geològic de Catalunya, Instituto Geográfico Nacional, Forest Property Centre, Montseny Association of Forest Landowners, Montseny Natural Park and Catalan Fire Department.

**Fig 6:**
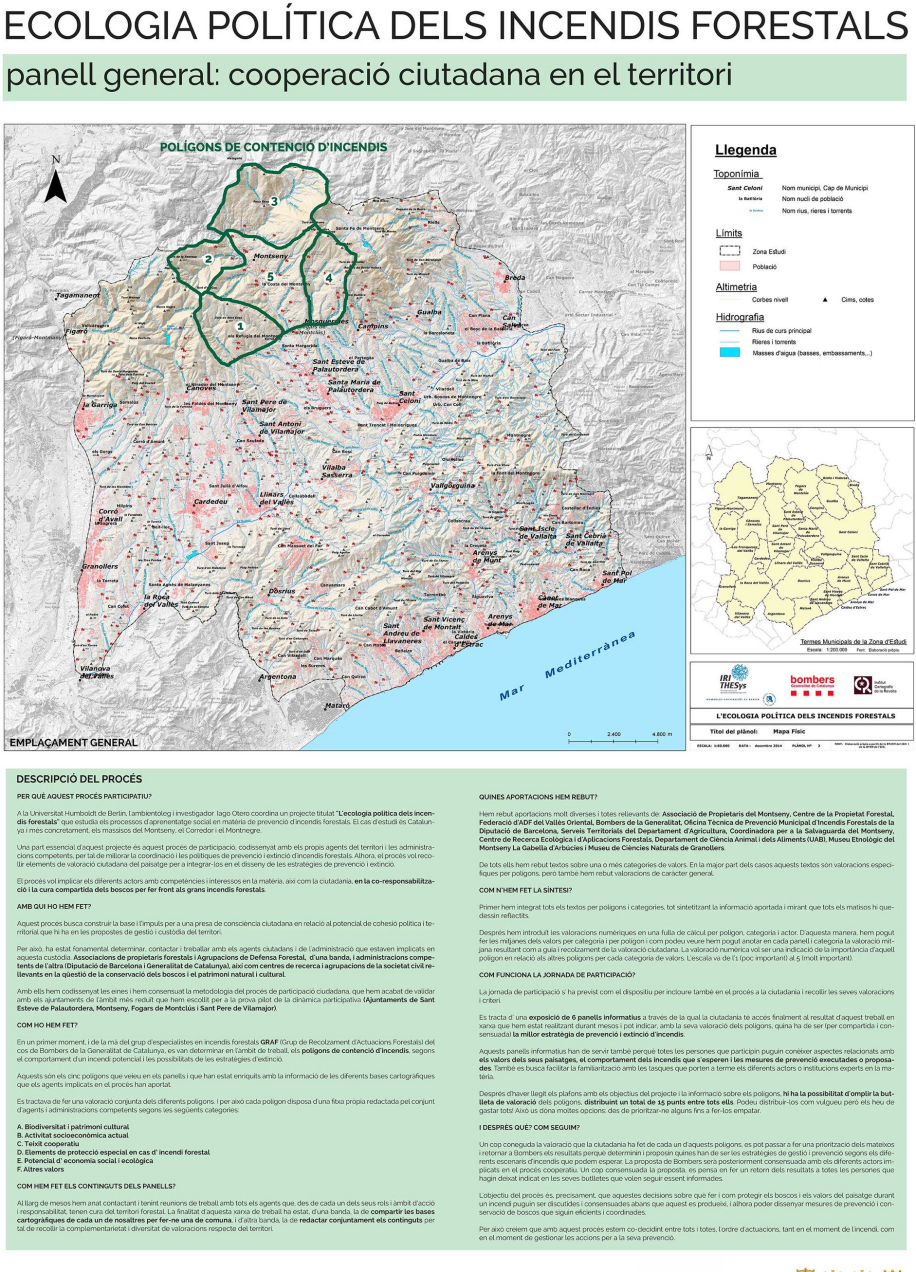
Poster used in the participatory exhibitions, containing general information on the project. The poster included the study region, goals, actors, method of landscape co-valuation in wildfire contention polygons, public participation procedure, and subsequent steps. Source: own elaboration based on input from the participatory process. The maps within the posters contain data from Institut Cartogràfic i Geològic de Catalunya, Instituto Geográfico Nacional, and Catalan Fire Department.

**Fig 7:**
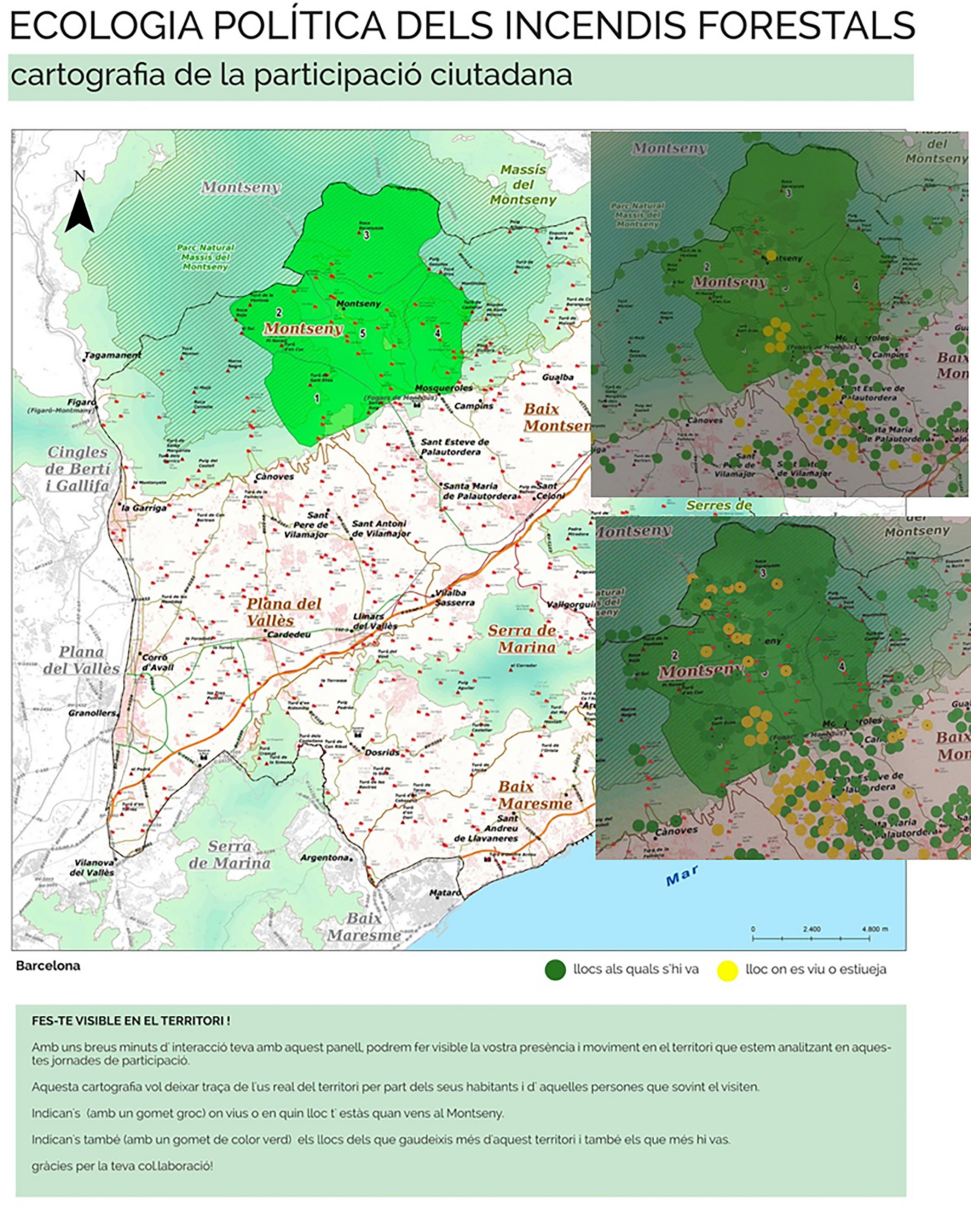
Interactive poster where the participants of the exhibitions were asked to attach a yellow sticker in their place of residence and green stickers in those places that they used (for shopping, leisure, etc.). On the right, we have superimposed pictures of this poster after the exhibition of 23–24 April (above) and 4 May (below). Source: own elaboration based on input from the participatory process. The maps within the posters contain data from Institut Cartogràfic i Geològic de Catalunya, Instituto Geográfico Nacional, Montseny Natural Park (zoning currently annulled) and Catalan Fire Department.

One poster per polygon was designed based on the landscape valuation synthesised from actors’ input (Fig 5). The synthesis text was shortened and edited for clarity. Each poster provided information on the different categories of landscape values and their relative importance as compared to the other polygons. The general average value of the polygons was not shown in the posters. Posters also included information on the wildfire and forest management tasks performed by each actor of the wildfire governance system so that citizens could learn about them. These posters were the basis of the citizen valuation exercise. Two additional posters were designed, one presenting the project (Fig 6) and another one asking participants to locate themselves in the study region by means of stickers (Fig 7).

A sheet was designed to gather participants’ valuation in the exhibitions (Table 6). The sheet included a numerical assessment that consisted in distributing 15 points among the 5 polygons according to their subjective relative importance. This gave the participants the option to prioritize polygons or to assign the same value (3) to all. In this way, those participants that would not necessarily agree on the need to prioritize polygons, based on ethical or practical reasons, could still participate. The sheet also included a qualitative assessment, i.e. the reasons for their particular distribution of points. It offered the possibility of adding any missing value, and asked the participants what they learnt from the exhibition. The sheet gathered basic personal data to monitor the composition of the voters.

**Table 6:** Sheet given to citizens attending the participatory exhibitions. Citizens were asked to fill in this sheet after reading the posters on landscape values prepared with the input from actors. Translated from Catalan. Source: own elaboration.

**Personal data** Name (optional): E-mail (optional): Would you like us to inform you about the next steps and the results of the project? Place of residence: Do you belong to an active organization in the study area? Which one?
**Valuation** Please distribute 15 points among the 5 polygons according to their importance for you. You must use all 15 points and no more than 15 points, but you can distribute them as you wish. P1□ P2 □ P3□ P4 □ P5□ Please explain us the reasons of your valuation: What did you learn from reading the posters in this exhibition? Would you like to add a value from the polygons that was not mentioned in the posters?
The Political Ecology of Wildfires Place: Date:

### 3.7. Conducting participatory exhibitions in the pilot area

Two participatory exhibitions open to all public were held in April and May 2016 in two towns of the pilot polygons (Figs 2 and 3). The two town councils co-organized the exhibitions with the facilitators and advertised them on their webpages and through their social networks. The exhibitions were also advertised through leaflets and personal networks of the facilitators. The actors involved in the previous stages of the process (group 1 and 2) were also invited to attend. The exhibition ran one and a half days in Sant Esteve de Palautordera and half day in Montseny. As participants showed up, the facilitators guided them through the exhibition and explained the goals of the participatory process. First we showed the introductory poster (Fig 6) and then the interactive one (Fig 7). Then we showed some slides explaining the logic of wildfire propagation across contention polygons and the role of SMP, as well as why a public landscape valuation was needed to inform strategies and prioritize interventions. Next we gave them the valuation sheet (Table 6) with instructions on how to fill it, and asked them to do so after reading the polygon-specific posters (Fig 5). For this, they were given unlimited time.

**Fig 8:**
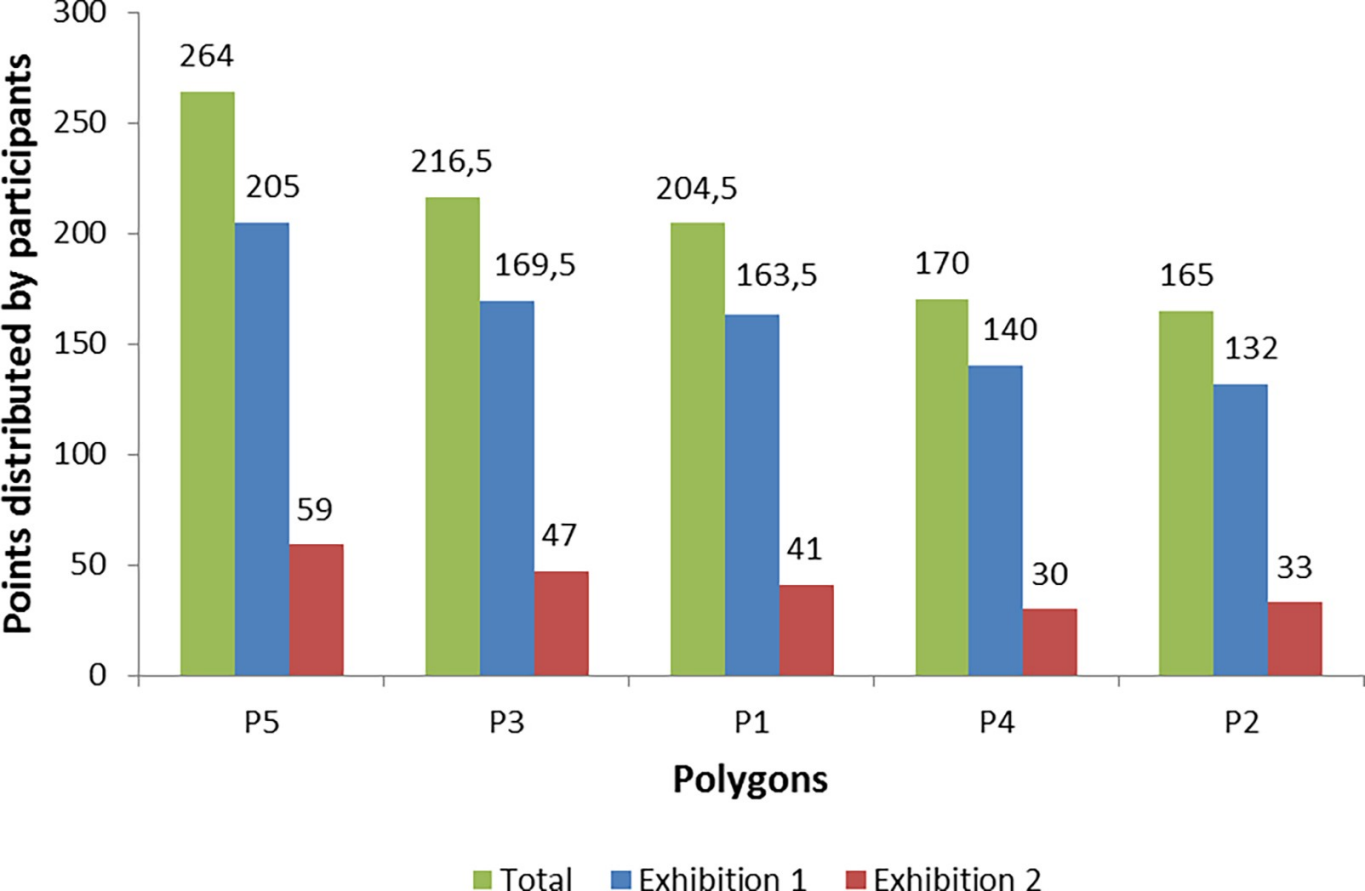
Results of the public prioritization of polygons (total, exhibition 1 and exhibition 2). Overall, 68 votes were casted, distributing 1020 points. Exhibition 1 occurred in Sant Esteve de Palautordera the 23^rd^ and 24^th^ of April 2016. 54 votes were casted, distributing 810 points. Exhibition 2 occurred in Montseny the 4^th^ of May 2016. 14 votes were casted, distributing 210 points. Source: own elaboration.

After the exhibitions the data from the valuation sheets was inserted into a spreadsheet. This had a row for each participant including personal data, distribution of points, and a summary of the qualitative information provided by him/her (reasons for the distribution of points, additional values and things learnt). Basic descriptors of the two sets of voters were created such as gender composition and place of origin. The points distributed by participants were added up per polygon. In the first town, 56 participants filled the valuation sheet, including the facilitators (4). One of the participants distributed a number of points other than 15 and another one chose not to provide a numerical assessment. Thus, 54 valid votes were collected. In the second town, 14 participants filled the valuation sheet. Overall, 68 persons voted, distributing a total of 1020 points. As shown in Fig 8, polygon #5 turned out to be the most valued one with 264 votes, followed by #3 (216.5), #1 (204.5), #4 (170) and #2 (165). The two participatory sessions yielded similar priorities, as there was disagreement only in the fourth and fifth positions (Fig 8).

### 3.8. Designing a wildfire strategy sensitive to social values about landscape

**Fig 9:**
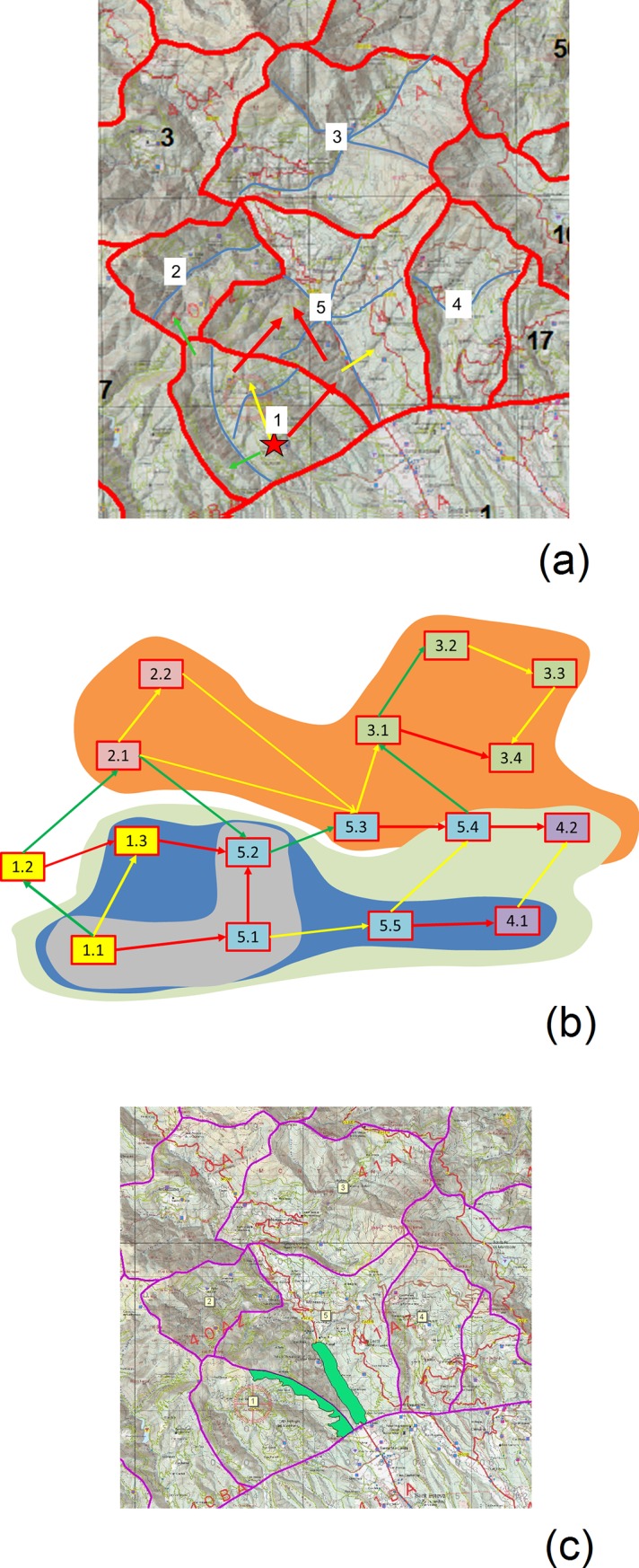
Summary of the wildfire strategy designed by GRAF and discussed with the actors. a) The strategy was prepared for a wildfire starting in polygon 1 (red star) and driven by west winds. Red arrow: front spread (high intensity); yellow arrow: flank spread (medium intensity); green arrow: back spread (low intensity); blue lines: wildfire contention sub-polygons, used to estimate the potential area burnt. b) Potential area burnt according to the success or failure of the strategy, as well as fuel conditions. Grey, blue and green areas correspond to a wildfire starting in sub-polygon 11 (discussed with the actors). The orange one corresponds to another starting point, which could eventually be discussed in future exercises. c) Strategic Management Points (SMP) that need to be developed to reach wildfire friendly landscape structures and make possible the strategy under discussion. Developing the SMP in polygon 5 would provide an opportunity for the Fire Department to contain the wildfire in the valley bottom. If this would work the burnt area would resemble the grey shape in Fig 9B. Instead, if this SMP is not developed, the burnt area would resemble the blue or green shapes. As a complement to this SMP, GRAF suggested to develop the SMP between polygon 1 and polygon 5 to reduce spotting distance and facilitate wildfire confinement in the grey shape. Source: own elaboration with data from Catalan Fire Department.

The results of the citizen valuation were then integrated in the design of a wildfire strategy sensitive to social values and priorities about landscape (Fig 3). Even if the polygons’ ratings were very close to each other, some priorities were evident. Sub-polygons were drawn in each polygon to better understand the spread patterns and the potential area burnt according to wildfire behaviour and strategy (Fig 9). The worst case scenario was used, i.e. a wildfire starting in sub-polygon 1.1 and propagating under west winds with all five polygons available to burn. The fire spread was drawn by distinguishing between front, flank and back propagation.

The suggested strategy consisted in working the back-left flank of the wildfire to avoid the propagation to sub-polygons 1.2 and 1.3, thus minimizing losses to the third most valued polygon. The opportunity to work on the front was instead considered to lie in the wildfire’s downhill move in sub-polygons 5.1 and 5.2. If the Fire Department would succeed in these two tasks, the wildfire would be contained in the area 1.1 + 5.1 + 5.2 (Fig 9B, grey area). This would save more than half of polygon 5 and all polygon 3, the two most valued by participants. If instead the opportunity in the front would be lost, the wildfire would propagate to other sub-polygons of 5 and to polygon 4 (Fig 9B, blue or green areas depending on whether the firefighters could work on the flanks or not). It is important to underline that in a high intensity wildfire, sub-polygons 1.1, 5.1 and 5.2 are considered to be beyond the extinction capacity, hence not workable.

To make this strategy possible, the implementation of two SMP was suggested (Fig 9C). The first one is located in the valley bottom at the centre of polygon 5 and falls within the municipalities of Sant Esteve de Palautordera, Fogars de Montclús and Sant Pere de Vilamajor. It would facilitate firefighting operations to avoid that the wildfire moves uphill to the opposite slope of the valley. For this, it was considered necessary to achieve a discontinuous vegetation structure preventing crown spread and thus limiting wildfire intensity so that the firefighters can work. The second SMP is located before the crest line dividing polygon 1 and 5, and falls within the municipalities of Sant Esteve de Palautordera and Sant Pere de Vilamajor. Lower fuel load in this SMP would slow down the wildfire’s uphill move and thus reduce spotting distance to polygon 5.

### 3.9. Discussing the strategy and the next steps with actors and participants

In May 2016 a joint meeting was held with group 1, 2 and 3 actors, where the participants of the exhibitions were also invited. First, we presented a summary of the participatory process, the composition of the two sets of voters and the quantitative and qualitative results of the citizen valuation. Next we presented and discussed the proposal of value-sensitive wildfire strategy and related SMP (Fig 9), which was agreed upon in the same meeting. Finally we identified some applications of the pilot project and discussed proposals for next steps (Table 7). Some of these applications stressed the interest of further developing the multi-actor GIS to coordinate interventions and adapt wildfire strategies to changing landscape structure and fuel conditions, while others emphasized that the project’s materials and network could be used as vehicles for knowledge dissemination on wildfire prevention and civil protection.

**Table 7:** Project applications and proposals to continue networking as discussed in the joint meeting with all actors in May 2016. Source: own elaboration.

Use the common GIS developed during the project to monitor management interventions and to adapt the Fire Department’s wildfire strategies to the landscape’s changing state. Create a virtual platform and give access to all actors so that current and future intervention proposals can be jointly operationalized.
Integrate selected strategic management points (SMP) in landowners’ forest planning schemes.
Use the project’s planning network as a vehicle of knowledge exchange in civil protection, for instance by disseminating information on self-protection to neighbours of vulnerable residential areas.
The Fire Department can protect landscape values as well as people and properties.
Implement the selected SMP with the collaboration of the different actors.
Involve the Catalan Department of Territory and Sustainability, responsible for urban planning, in subsequent steps of the project. Incorporate existing planning schemes in the common GIS to check obstacles and opportunities for the integration of wildfire risk into urban planning.
Organize a specific meeting with the mayors of the municipalities included in the 5 pilot polygons to discuss the continuation of the project at the local scale.
Suggest to those mayors that they present a more concrete version of the project proposals to the board of the Natural Park and Biosphere Reserve of Montseny.
Present the project experience in the board of the Forest Property Centre, composed by representatives of different Departments of the Catalan government.
Expand the project by replicating the pilot valuation to all Montseny mountains. The mayors of the pilot municipalities may invite the rest of the mayors to join such a project.
Disseminate knowledge by circulating the posters used in the participatory exhibitions around different places in combination with dissemination efforts by the Fire Department and the Federation of Forest Defence Associations.
Distribute a copy of the posters used in the participatory exhibitions among the project´s network of actors and citizens.

The actual implementation of the agreed upon SMP was suggested, but a consensus emerged that the mayors of the 4 municipalities involved in the participatory exhibitions should take the lead in the continuation of the project. Several actions to do so were suggested, such as inviting the rest of the mayors of the Montseny mountain ranges to join in a second phase (Table 7). In the meeting, the project was in general considered a first step towards a Fire Department that is able to protect landscape values as well as people and properties, and it was deemed interesting to present the experience to the boards of some of the involved institutions. It was considered crucial to involve experts and policy-makers in land and urban planning to integrate wildfire risk into planning schemes at different scales.

Finally, a meeting was held in June 2016 with the mayors, councillors and heads of local forest defence associations to discuss the intervention proposals relevant at the municipal and multi-municipal scales (Table 8). It was organized by the mayors following one of the agreements of the previous joint meeting (Table 7). It was considered necessary to share the cartographic information on SMP and the contact details of participants for coordination purposes, as well as to set up a virtual platform with the multi-actor GIS accessible to the town councils’ technical services. The organization of dissemination activities on wildfire prevention for vulnerable residential areas was suggested, and the mayors committed to requesting a meeting with the Natural Parks division of the Barcelona Province Authority to budget the implementation of the priority SMP. To ensure the long-term economic feasibility of maintaining appropriate forest structures in the SMP, it was considered that the firewood could be used to supply biomass boilers already existing in municipal equipment facilities for heating, while creating links with the biomass sector in the region.

**Table 8:** Some agreements and proposals discussed with the mayors and other group 3 actors in the final meeting of the project. Source: own elaboration.

The mayors request a meeting with the Natural Parks division of the Barcelona Province Authority to ask financial and technical support to design and implement the priority strategic management points (SMP).
GRAF transfers the GIS with the SMP to the town council’s technical services.
ICR makes a budget to set up the virtual platform with the common GIS, with access to all participants.
The firewood resulting from the implementation of the priority SMP can be used to supply existing biomass boilers in municipal facilities. Income from firewood can be invested in the maintenance of the SMP. Collaboration with the Montseny Association of Forest Landowners and a nearby biomass cooperative could invigorate the region’s biomass sector.
Several dissemination activities can be co-organized by the town councils and the Fire Department on prevention and self-protection, for residents in vulnerable developments.
IRI THESys disseminates a press release on the project results.

## 4. The participatory process: Democratic and transformative wildfire planning?

### 4.1. Position and legitimacy. Participating in what?

GRAF was both an agenda setter and an invited actor in the participatory process. This situation stemmed from the first author’s long-term collaboration with GRAF in wildfire research and management projects. Being sympathetic to GRAF´s vision on wildfires, the first author had to perform a “neutral” role of facilitator among actors while “siding” with one of them. This contradictory position raised important questions about potential biases in the design and implementation of the participatory process. We controlled for these biases by i) characterizing the challenge, ii) implementing the co-design principle, and iii) reflecting on the actual reach of actors´ and citizens’ participation.

The challenge was to democratize GRAF’s decision power over the region’s landscape by means of a participatory method that was both operational, i.e. translatable to concrete wildfire strategies, and legitimate, i.e. integrating the values and management tasks of other actors in a way that was meaningful for them. For this, we implemented the co-design principle, based on reaching agreements with actors on what was to be done and how during the entire process, from the initial individual meetings to the final decisions about how to continue after the pilot phase. This required flexibility to adapt the process’ development to actors’ changing engagement in it. The landscape valuation method was for instance co-designed with actors and tested in joint meetings, including what to value and how, contradictions between values, whether prioritization of polygons was possible at all and which criteria should be used for this. The process likewise accommodated actors’ attempts to turn it into something useful for their interests, something that implied building alliances with them. For instance, the forest owners saw the process as a potential tool to enhance forest management, disseminate their role as land stewards, better allocate public funding for forest management and simplify administrative procedures. For the Forest Property Centre, the results of the participatory process could be used to prioritize public funding and to negotiate with forest owners on special management conditions required to implement the strategic management points (SMP). The Barcelona Territorial Service of the Department of Agriculture was interested in the process as a way to improve inter-agency coordination. The process fit in the general interest of both Natural Parks to improve governance through more democratic decision-making bodies. For the Technical Office of Municipal Wildfire Prevention of the Barcelona Province Authority, the process could be an opportunity to better establish which interventions in private estates are financed with public money and which ones with private money.

A clear example of how actors shaped the process according to their own interests–legitimizing it along the way–is the organization of the second participatory exhibition, which was not planned by the facilitators but proposed by a councillor of Montseny municipality. When he attended the first exhibition–occurring in a town of the plain–he invited us to bring it to Montseny–a mountain municipality–and took charge of the dissemination. The board meeting of the local forest defence association was scheduled immediately after the exhibition and in the same venue so that its members could participate and vote. Not surprisingly, this participatory session turned out to be mostly composed of mountain settlers and members of the forest defence association. As the poster locating participants’ residence was filled with yellow stickers in the mountain areas (Fig 7), the councillor told us “You see? Now it is balancing”. Even if the councillor’s move could be read as an attempt to lobby the process, it turned out to balance it in terms of participants’ place of residence and sociological profile (Section 4.2).

The creation of a common GIS made the co-design principle visible and believable for the actors. When forest owners said that GRAF’s wildfire contention polygons were not meaningful for them, the facilitators decided to ask all actors those GIS layers representative of their wildfire and forest management tasks. A GIS was subsequently built with layers from almost all group 1 actors including private and public properties with forest planning schemes, wildfire and landscape management interventions by forest owners, forest tracks and water infrastructures maintained by public agencies, and zoning of natural protected areas (Fig 4). These layers were overlapped with GRAF’s (Figs 2 and 4B). In the first joint meeting with the actors, the GIS layers were displayed, commented by each actor and discussed to highlight common interests and potential synergies. The actors, including GRAF, could explain their needs and expectations in a transparent way. This served to recognize the existing diverse perspectives on wildfire and forest management, while stressing–and legitimizing–the need of synthesizing them at the scale of wildfire contention polygons to inform the strategies of the Fire Department.

The above leads us to reflect on the actual participation of actors and citizens in the process. They co-valued the landscape and prioritized polygons to inform the Fire Department’s strategy, which included choosing among a set of initially defined SMP. Yet they did not participate in the production of knowledge about wildfires and how they should be fought. The role of wildfire experts was assigned to the Fire Department and, in particular, to GRAF, whose wildfire contention polygons and SMP framed the valuation exercise from the beginning. Even if the polygons and the need to prioritize according to social landscape values were intensely discussed with actors in the first joint meeting, GRAF’s technical knowledge and overall framing were not contested, reproducing its hegemonic position in the Catalan wildfire governance system [1]. However, while the participatory process did not question GRAF’s knowledge, it opened expert wildfire management to society by incorporating landscape values into the Fire Department’s strategies.

Indeed, the participation of actors and citizens had large implications. Opening GRAF’s decision-making power to wildfire governance actors, landscape inhabitants and landscape users, was not always easy. Some actors agreed to help the Fire Department to make more informed decisions during wildfires, but still stressed that it is this agency which is responsible for these decisions. In the participatory exhibitions, citizens spent a long time reading the posters and thinking how to distribute the points in the valuation sheet. This suggests that they were not only aware of the complexity of the valuation but also of the implications of their participation for actual decision making. However, making an informed decision required time and also the capacity to process multidimensional information. Some participants, for instance, complained about not having had enough time to read the posters. It is thus likely that in many cases the valuation was done after assimilating only a share of the information provided. Moreover, the ways in which the participatory landscape valuation would be translated into a concrete wildfire strategy were not well communicated because at the time we did not know how such a translation would be done.

In the end, the aggregated numerical assessment (Fig 8) turned out to be the only criterion used to design the wildfire strategy. Neither the qualitative valuation and the additional values reported by the participants nor the values and management proposals provided by the actors in the posters had a direct translation into the strategy. Moreover, even if polygon #5 turned out to be the most valued one, the Fire Department committed to save only half of it, arguing that under a high intensity wildfire the other half was not workable for the firefighters (Fig 9). This suggests that the capacity of participants to decide how a wildfire affects their landscape was in actual fact limited. The feedback meeting explaining how the valuation had been translated into a strategy was thus important to round off the exercise. Still, future exercises should provide clearer information on how the valuation will be translated into concrete strategies, including the technical limits of extinction.

### 4.2. Who participated in the process and who did not?

All the actors that we considered part of the wildfire governance system (Table 2) participated in the process except the Federation of Forest Defence Associations of the Maresme County, which turned out to be of less importance as the pilot polygons were located in the Vallès Oriental County. However, some institutions with competencies in wildfire governance were not included in our sketch of the wildfire governance system. One of them was the Civil Protection Authority of the Department of Home Affairs, which is in charge of drafting wildfire emergency plans that coordinate the different agencies involved when a wildfire takes place (INFOCAT plan). In our study region, some of this Authority’s competencies are de facto transferred to the Technical Office of Municipal Wildfire Prevention of the Barcelona Province Authority, which supports municipalities in prevention and emergency planning. As this agency was included in our process, the non-participation of the Civil Protection Authority did not necessarily entail that its role in wildfire governance was neglected. Another institution that was not included was the Service of Forest Rangers of the Department of Agriculture, which function (scientific investigation of wildfire causes) we considered less relevant for the purposes of our process. The Urban and Land Planning Authority of the Department of Territory and Sustainability was also not included, despite the fact that it could become a key actor promoting the integration of SMP into legally binding urban and land planning schemes. The need to invite this agency was identified by the participants of the process, but the facilitators were not able to achieve this. As these three agencies were not present in the participatory process, the outcomes of our project could eventually be less relevant for them.

Beyond the wildfire governance system, wildfire risk is influenced by and influences a wide array of actors. Representatives of infrastructures crossing our study region (AP7 highway, international high-speed train, conventional train and electric lines) as well as industrial and touristic activities occurring in the region were not included in our process due to time and resource constraints. Similarly, the representation of potentially affected interests outside our study region due to a cut in infrastructures after a wildfire event inside our study region was not deemed feasible, but should be considered in future processes.

Actors participated to varying degrees ranging from only attending the meetings to actively contributing to the landscape co-valuation. In the test phase we got input from 6 out of the 10 invited actors from group 1. In the pilot co-valuation we got input from 13 out of 15 actors from group 1 and 2 (excluding those with a focus on Montnegre-Corredor). As actors had overlapping interests and expertise, contributing actors partially compensated for the missing information from non-contributing actors. Redundancy across actors was thus important to avoid neglecting certain landscape values. Still, redundancy was not a guarantee that all potentially relevant information was considered. The Natural Park of Montseny, for instance, did not provide any input on landscape values. This was partially compensated by the biodiversity assessment provided by the Museum of Natural Sciences in Granollers and the information on cultural heritage provided by the Montseny Ethnology Museum in Arbúcies, showing the usefulness of inviting a set of additional actors (group 2). However, the distribution of an endemic amphibian species of high conservation value (Montseny brook newt, *Calotriton arnoldi*) was for example missing because the Granollers expert on amphibians was not in the internal meeting where the assessment was drafted.

The participatory exhibitions attracted primarily residents of the municipalities were they were held (29% of participants of exhibition 1, E1, and 57% of participants of exhibition 2, E2). Even if participants also came from the other municipalities included in the pilot polygons (13% of E1 and 7% of E2), the two municipalities that did not hold exhibitions (Sant Pere de Vilamajor and Fogars de Montclús) were underrepresented. Exhibitions also attracted participants from other municipalities in the Montseny region, outside the pilot polygons (38% of E1 and 29% of E2) and from Barcelona city (13% of E1), entailing that the local landscape valuation was partly done by extra-locals. An important share of participants was formed by mayors and councillors, mostly from the municipalities in the pilot polygons (16% of E1 and 29% of E2).

The two sets of voters were qualitatively different. The first one was mostly composed of residents in the valley bottom, was gender balanced, had a significant share of anti-capitalists and ecologists (13%), and a relatively low share of participants linked to local forest defence associations (11%). In contrast, the second sample was mostly composed of mountain residents including forest owners, was male dominated (86% of the participants), and had a relatively high share of participants linked to forest defence associations (50%). The sociological composition of both sets of voters was conditioned by the dissemination. The first one was mostly done by I.O., who attracted his activist network, and by two co-organizing town councils through their social networks, excluding people with no access to these communication tools. The second one was instead done by the hosting town council through letters to neighbours and the board of the local forest defence association. In both cases dissemination did not reach all residents in the four municipalities. Despite differences in sociological composition, the landscape valuation in the two exhibitions was surprisingly similar (Fig 8).

### 4.3. Valuing landscape and learning

A number of questions emerge around what was valued, by whom, and how. Regarding the first point, the content of the valuation was contingent upon the actors included and their availability to provide input. As such, it was necessarily non-exhaustive and relevant values potentially influencing public priorities could have been left out, exemplified by the endemic amphibian species. Regarding who defined the value categories, categories such as ‘Biodiversity and cultural heritage’ or ‘Current socioeconomic activities’ were created based on the actors’ input during the test, while others such as ‘Cooperative fabric’ and ‘Potential for social and ecological economy’ were created by the facilitators to give the process a transformative direction (Table 4). The citizens participating in the two exhibitions had no influence over the value categorisation, and their function was limited to the prioritization of polygons based on the qualitative and quantitative information provided by the aforementioned actors.

Regarding how landscape was valued, heterogeneous methods and criteria were used by the participants in the process. While some actors used numbers to assess the relative importance of polygons during the co-valuation, others provided only texts. The ranking of polygons differed between group 1 and group 2 actors (Table 5), although we did not check whether these differences were statistically significant. Similarly, the citizen valuation changed the prioritization of polygons done by actors, but not dramatically (actors: P5>P3>P4>P1>P2; public: P5>P3>P1>P4>P2). In the participatory exhibitions, citizens reported to use a diversity of criteria to distribute the points. These included biodiversity, aesthetics, emotional, private property, wildfire propagation, presence of houses and developments, potential for social and ecological economy, cultural heritage, as well as unwillingness to prioritize. Additional values reported by citizens included forest type and age, a public rural school, the regenerative potential of the land, intervention proposals such as silvopasture or biomass production for energy, threats due to development projects, immaterial cultural heritage on fire, an ancient tree, and the endemic amphibian that was left out in the co-valuation. Ranking polygons based on their aggregate score thus synthesized heterogeneous and potentially contradicting values into a collective prioritization to inform the wildfire strategy. However, the use of a numeric synthesis of collective priorities may have detracted from more qualitative discussions of values and policy goals, even if qualitative information was prominent in the posters and deliberation on policy proposals occurred in the meetings.

In the valuation sheet, citizens participating in the exhibitions reported to have acquired more and better knowledge on a wide diversity of landscape values as a result of being involved in the exercise, sometimes increasing their awareness of the environmental importance of the areas where they live or spend leisure time. Several participants stressed the interest of learning to value the landscape from the perspectives of different actors. They reported to have learnt the existing wildfire types and the logic of wildfire spread and contention polygons. They also learnt how the Fire Department develops the wildfire strategies, as well as the relevance of the wildfire prevention and forest management tasks developed by those actors linked to forest owners (local forest defence associations, the Montseny Association of Forest Landowners and the Forest Property Centre). Participants reported to have learnt wildfire prevention and land management proposals too, including the recovery of pastures and cropland, the importance of forest and territorial planning, and the need of a multi-actor planning of wildfire prevention policies that integrates diverse criteria and social interests about landscape. Some participants learnt that protection from wildfires can enhance economic activities and that the ways in which society lives with nature shape wildfire risk. All these were learnings that we wanted to enhance with the participatory exhibitions. We also wanted to disseminate the idea that wildfire is a part of the ecosystem that can be managed for positive ends, and that wildfire prevention entails a re-organization of human-environmental interactions, but according to the feedback gathered in the valuation sheet these insights did not reach the participants.

### 4.4. Implementing the outcomes of a pilot process?

Ideally, to implement the wildfire planning proposals stemming from our process, the call for participation should have reached 100% of the electoral roll by official means, and the decisions made should have been approved by the town councils’ plenary. Still, given the efforts made and the high wildfire risk in the region demanding urgent interventions, trying to implement the chosen SMP regardless was tempting. But when interventions had to be agreed upon, some difficulties emerged. The Technical Office of Municipal Wildfire Prevention of the Barcelona Province Authority refused to fund coordinated measures. Underlying this decision was the conflict between the office and the Montseny Association of Forest Landowners, which unlike the one in Montnegre-Corredor, does not collaborate in the office’s forest planning for wildfire prevention (Table 3). The Montseny forest owners instead stressed the need to implement the SMP, which they considered legitimate enough, to reduce wildfire risk. Other actors warned that this would require building further legitimacy, with mayors taking the lead and conducting a more systematic public participation program. An agreement seemed to emerge between the two positions: the SMP could be implemented in parallel to new efforts to increase the democratic content of the participatory process. After the last meeting, the facilitators retired from their leading roles and observed the endogenous dynamics generated by the process. After 2 years, the mayors have not carried on the work necessary to implement the wildfire planning proposals, but the forest owners have held some preparatory meetings to do so.

### 4.5. Conflict, consensus and the re-framing of the wildfire issue

In our meetings, it was evident that the invited actors’ framing of the wildfire issue went beyond the traditional suppression-based approach and instead thought of it as a matter of forest and landscape management. The participants of the exhibitions likewise learned that wildfire prevention is linked to a number of land management proposals such as silvopasture. However, during the process there was not enough reflection on which politic-economic policies are necessary to make such management proposals possible in a largely urban and industrial context where agro-silvo-pastoral activities are not profitable anymore. The Coordination Group for Montseny Defence stressed in the debates that recovering rural activities in the mountain required facilitating young people access to land and debating about the concept and laws of land property. This was an opportunity to reframe the wildfire issue in politically transformative ways that was however not given sufficient attention by the facilitators. Indeed, as the networking with the practitioners of alternative forest and land management practices failed, there was not a critical antagonist mass to reframe the wildfire issue as a problem of political-economic and land-use model that needs to change if we are to build a resilient landscape.

Instead, the debates were mostly chaired around the need to inform the strategies of the Fire Department through landscape co-valuation, and how to operationalize it. For this, consensus among actors was always reached. Consensus was however not only due to our facilitation, but built on previous collaborative wildfire prevention projects between some of them. Conflictive issues within the wildfire governance system, such as the integration of local forest defence associations in the Fire Department’s suppression operations, surfaced during debates on valuation but never derailed the consensus. Existing conflicts between actors did not play out strongly in the co-valuation exercise or in the citizen valuation–but interestingly did so when interventions had to be agreed upon.

The above suggests that in future processes a better identification and management of potential conflicts and disagreements among participants may be advisable to strengthen the transformative power of the network, both to articulate potentially re-framing visions and to ensure that the process’ outcomes can be actually implemented on the ground.

## 5. Discussion

Various papers have emphasized the potential of participatory planning networks to build wildfire resilient landscapes in the face of current and future impacts of suppression and land-use and climatic changes [3–7]. Studies have stressed the need to understand resilience as a process of adaptive governance mediated by institutions at multiple scales, as it opens social opportunities to learn from and adapt to wildfire [11,46]. In turn, building resilient landscapes has been shown to require fundamental transformations in social-ecological relationships [47,48], including fossil-fuel based food and energy systems and the ensuing high risk landscapes [1]. For this, authors have stressed the need to create sites of “healthy contestation” whereby the political economy of land use is opened to debate and antagonism is channelled into transformative avenues [20,49,50]. The creation of “political communities” able to deal with conflicting values while shaping their own fire regimes has been likewise suggested as a way forward [8,9].

Our experience shows how a planning network incorporating landscape values and local knowledge into operational wildfire strategies can be designed and implemented in a fire-prone region. By planning and developing links between academic and non-academic actors across scales, the pilot project also illustrates the potential of action-research [35,36] and transformative project co-design [33] for building resilience through the setup of ad-hoc political communities, as well as some of the main challenges ahead.

Our method complements existing approaches that incorporate social values about landscape and local knowledge into operational wildfire management plans and decision support systems [16–18]. Our prioritization of polygons based on an aggregate score offers a relatively simple way to translate complex social values into concrete wildfire strategies. In turn, the spatially explicit representation of personal values developed by [18] could be used in a follow-up of our pilot process, in order to also translate participants´ qualitative valuation and management proposals into more inclusive wildfire strategies. Interestingly, we did not find big disagreements on social priorities between two very different sets of citizens, something that has been also reported in similar experiences [18]. This suggests that the conflicting nature of wildfire policies reported by some studies [20,51–55] might not necessarily hold in the particular case of wildfire planning networks, where basic consensus seem to emerge around what is to be protected first. Future efforts should therefore explore whether this consensus also exists when wildfire strategies are democratized beyond the pilot (micro) scale.

Our pilot process shows that the exclusion of relevant actors, citizens and values was not only due to the practicalities of the project, but also to the extremely broad range of interests potentially affected by the large wildfires expected in the region. This fact points at a fundamental trade-off between high wildfire risk levels and democracy that should be considered in further efforts to democratize wildfire management [9]. Our experience however suggested some offsetting mechanisms, including value redundancy across invited actors, and a continuous improvement of the network´s democratic content while the (imperfectly) agreed upon interventions are implemented. This in turn requires resources and incentives to continue networking and to monitor the interventions on an ongoing basis [17], something that is not always provided by the institutional arrangements and funding mechanisms of universities or public agencies [56]. Indeed, when our project ended the network hardly continued its activities, suggesting that more efforts should be devoted to secure the follow-up of pilot processes [18]. A key follow-up stage should involve the creation of the actual decision support system including the multi-actor GIS, the valuation results and the agreed strategy and interventions. Using these elements in combination with tools that facilitate stakeholder´s deliberation about alternative fire management policies [9,11] could potentially enhance the network’s transformative power.

Our experience reveals how the connection of different governance scales through a wildfire planning network (re-)defines agency over landscape, i.e. the power to actively shape land-uses, values and wildfire impacts while building resilience. Priority interventions were defined at the local level by both local and extra-local citizens and actors. In turn, the ability of citizens to define the wildfire strategies that would transform their living environments for decades was constrained by suppression limits and by the participatory method itself. Therefore, the positive link between local empowerment and better wildfire governance reported by some studies [8,17] should be problematized according to the varying ways in which planning networks are operationalized across scales. Rather than considering local agency and bottom-up processes as the privileged realm for transformative action, our experience suggests that wildfire planning networks could benefit from a strategic mobilization of the knowledge available at different scales [57]. Indeed, the combination of top-down and bottom-up valuation mechanisms gave our process structure and direction while leaving space for autonomous development and learning, as it has been found in other collaborative planning contexts [58]. These insights suggest that participatory and transformative planning networks may offer the kind of adaptive governance structures and processes needed to effectively deal with wildfire risk in the face of climate and land-use changes [11,46].

The pilot project also illustrates how participatory planning networks might change the relationship between expert knowledge and agency over landscape as the wildfire strategies are democratized. As explained in our critical reflection on the participatory dynamics, GRAF knowledge was not negotiated and framed the whole process. But our participatory design facilitated a negotiation on the landscape effects of such knowledge–so far only in GRAF´s hands–via agreeing on a wildfire strategy that incorporates social priorities about landscape. In turn, the fact that citizens reported to have acquired some knowledge on wildfire and wildfire management from GRAF and other agencies suggests that expert agencies can enhance the recovery of local fire knowledge through participatory planning networks. Recovering fire knowledge by re-incorporating fire use in agricultural or grazing activities of local communities is at the heart of calls for innovative wildfire management [59]. This is however a challenging task given that traditional fire knowledge systems declined or vanished with industrialization [21]. Indeed, our process took place in a metropolitan setting where local communities have very limited experience in fire management. In the Barcelona metropolitan region and in many urbanized fire-prone regions of western societies, the Fire Department´s wildfire specialist groups can therefore play a key role as revitalizers of fire knowledge. Thus, a better understanding of how planning networks reconfigure traditional fire knowledge and how this reconfiguration affects community resilience and power relationships should be the object of further research [60].

Finally, our approach situates participatory wildfire planning networks in the broader framework of transformations to sustainability. Various papers have argued that addressing sustainability challenges requires a deliberate transformation of the systems that perpetuate environmental problems–minor adjustments in governance or institutions will simply not suffice [31–34,61,62]. Similarly, the attempt to coexist with wildfire has been shown to entail a radical social-ecological transformation of land-uses, settlement patterns, energy supply systems and social values about wildfires [1]. Our pilot process reveals that legitimacy and a suitable management of conflict/consensus are two key aspects underpinning the transformative capacity of a participatory wildfire planning network. The mechanisms by which our process built legitimacy among actors made possible the successful articulation of heterogeneous interests into a common purpose, i.e. informing the strategies of the Fire Department. This entailed opening the decisions shaping wildfires and landscapes from the techno-managerial up to the political realm. However, the unsuccessful articulation of actors with the potential to re-frame the wildfire issue also revealed the network´s inability to rethink wildfire resilience from a management problem to a political process whereby more sustainable social-ecological configurations are *deliberately produced* [30,63]. This suggests that the debate about the ideal distribution of responsibility in risk management across governments, communities and the private sector [64] should be expanded to encompass the distribution of transformative agency across social actors. By shedding light on the political-ecological processes in which planning networks are embedded and on their transformative potential, these insights can complement new planning approaches aimed at integrating the biophysical and social dimensions of wildfire risk [4,10,11,59]. Our method can also be used to operationalize fire restoration calls [24,25,65–67] by agreeing upon which wildfires will be left to burn, under what circumstances and according to whose values within the affected communities. Our experience suggests that the effectiveness of both endeavours will depend on their ability to build legitimacy across scales and to channel social and political conflict into transformative avenues.

## 6. Conclusions

In this paper we presented a method to democratize wildfire strategies by incorporating social values about landscape in suppression and prevention planning. Based on a pilot project, we described how this can be operationalized by developing a network of researchers, practitioners and citizens across spatial and governance scales. We showed that such networks have the potential to build resilient landscapes as they facilitate discussions and agreements on strategies that reduce expected wildfire intensity and minimize the loss of social values. The outputs of these networks can likewise contribute to improve wildfire risk maps by integrating social priorities with biophysical data.

Our paper pointed at several challenges for the successful implementation of such networks, and suggested ways to address them. Practical challenges include how to develop more inclusive social valuation methods and how to overcome institutional obstacles to long-term engagement in trans-disciplinary projects. Deeper challenges were shown to be related to the risk levels derived from the region´s current social-ecological setup. A trade-off between high wildfire risk and democratic wildfire management was suggested. In turn, the political negotiation on the landscape effects of expert knowledge was shown as a potentially transformative pathway that reduces risk, fosters re-learning on fire, and re-defines agency over landscape.

Our experience thus illustrates how democratizing wildfire strategies ultimately entails co-shaping the landscapes and societies of the future. The prefix “co-”refers both to a human collective involved in such deliberations and to wildfire itself. Given current risk levels, how to turn the latter from an enemy to an ally in the production of a new social-ecological setup should be given utmost attention by researchers, practitioners and policy-makers. Our method could eventually be used to prevent tragic wildfires while addressing–and correcting–their underlying social-ecological causes. However, as similar experiences are conducted beyond the pilot level and deeply antagonistic interests become involved, the challenges identified here will become ever more relevant. We therefor recommend that the projects’ goals, biases, design criteria, methodological choices and public prioritizations are kept open to successive phases of monitoring and deliberation.

## Supporting information

S1 Table Timeline of the participatory process. (DOCX)
