# Supplementary material for: Democratizing wildfire strategies. Do you realize what it means? Insights from a participatory process in the Montseny region (Catalonia, Spain)
Source: PLoS One. 2018 Oct 16;13(10):e0204806. doi: 10.1371/journal.pone.0204806 (PMC6191092; doi:10.1371/journal.pone.0204806)
Supplement: S1 Table — (DOCX) [file pone.0204806.s001.docx]

**S1 Table. Timeline of the participatory process.** We outline the main tasks, the responsible (in brackets), and the month(s). For simplification purposes, we don’t show internal meetings of the core team, communication between the team and the actors, and management and budget tasks. GRAF: Support Group for Forest Interventions, Fire Department, Department of Home Affairs, Catalan regional government (Spain); IRI THESys: Integrative Research Institute on Transformations of Human-Environment Systems, Humboldt University of Berlin (Germany); ICR: Association Cartographic Institute of Revolt. See Table 3 for a description of group 1, 2, and 3 actors.

|  | 2014 | | | | 2015 | | | | | | | | | | | | 2016 | | | | | |
| --- | --- | --- | --- | --- | --- | --- | --- | --- | --- | --- | --- | --- | --- | --- | --- | --- | --- | --- | --- | --- | --- | --- |
|  | IX | X | XI | XII | I | II | III | IV | V | VI | VII | VIII | IX | X | XI | XII | I | II | III | IV | V | VI |
| Drafting wildfire contention polygons based on the region’s wildfire patterns (GRAF). |  |  |  |  |  |  |  |  |  |  |  |  |  |  |  |  |  |  |  |  |  |  |
| Mapping the wildfire governance system (continuously updated hereafter) (IRI THESys). |  |  |  |  |  |  |  |  |  |  |  |  |  |  |  |  |  |  |  |  |  |  |
| Actor-specific meetings with group 1 actors to explain the project, involve them and request their GIS layers (IRI THESys and ICR). |  |  |  |  |  |  |  |  |  |  |  |  |  |  |  |  |  |  |  |  |  |  |
| Mapping alternative forest and land management projects (IRI THESys). |  |  |  |  |  |  |  |  |  |  |  |  |  |  |  |  |  |  |  |  |  |  |
| Joint meeting with group 1 actors to explain the project, show overlap of actors’ GIS layers, and discuss a landscape co-valuation method (IRI THESys and ICR). |  |  |  |  |  |  |  |  |  |  |  |  |  |  |  |  |  |  |  |  |  |  |
| Gathering and synthesizing input on landscape values from group 1 actors in a test of 2 non-contiguous polygons (IRI THESys). |  |  |  |  |  |  |  |  |  |  |  |  |  |  |  |  |  |  |  |  |  |  |
| Actor-specific emails to group 2 actors to explain the project and involve them (IRI THESys). |  |  |  |  |  |  |  |  |  |  |  |  |  |  |  |  |  |  |  |  |  |  |
| Joint meeting with group 1 actors to discuss the synthesis of landscape values in test polygons and the landscape co-valuation method for citizen participation (IRI THESys and ICR). |  |  |  |  |  |  |  |  |  |  |  |  |  |  |  |  |  |  |  |  |  |  |
| Joint meeting with group 2 actors to explain the project and involve them in the landscape co-valuation (IRI THESys and ICR). |  |  |  |  |  |  |  |  |  |  |  |  |  |  |  |  |  |  |  |  |  |  |
| Design of landscape co-valuation method for 5 contiguous pilot polygons based on test input and joint meetings (IRI THESys). |  |  |  |  |  |  |  |  |  |  |  |  |  |  |  |  |  |  |  |  |  |  |
| Gathering and synthesizing input on landscape values from group 1 and group 2 actors for the 5 pilot polygons (IRI THESys). |  |  |  |  |  |  |  |  |  |  |  |  |  |  |  |  |  |  |  |  |  |  |
| Specific meetings with mayors of the 4 municipalities included in the 5 pilot polygons. Explanation of project and co-involvement in the organization of participatory exhibitions (IRI THESys and ICR). |  |  |  |  |  |  |  |  |  |  |  |  |  |  |  |  |  |  |  |  |  |  |
| Joint meeting with mayors, councillors and heads of forest defence associations of the 4 municipalities included in the 5 pilot polygons. Explanation of project and participatory exhibitions (IRI THESys). |  |  |  |  |  |  |  |  |  |  |  |  |  |  |  |  |  |  |  |  |  |  |
| Preparation of panels for participatory exhibitions (ICR). |  |  |  |  |  |  |  |  |  |  |  |  |  |  |  |  |  |  |  |  |  |  |
| Participatory exhibitions (ICR and IRI THESys). |  |  |  |  |  |  |  |  |  |  |  |  |  |  |  |  |  |  |  |  |  |  |
| Draft of wildfire strategy that integrates social values about landscape (GRAF). |  |  |  |  |  |  |  |  |  |  |  |  |  |  |  |  |  |  |  |  |  |  |
| Joint meeting with actors in group 1, 2 and 3 to discuss and approve the wildfire strategy, project applications and intervention proposals (ICR, IRI THESys and GRAF). |  |  |  |  |  |  |  |  |  |  |  |  |  |  |  |  |  |  |  |  |  |  |
| Joint meeting with group 3 actors to discuss intervention proposals at the municipal scale (IRI THESys and GRAF). |  |  |  |  |  |  |  |  |  |  |  |  |  |  |  |  |  |  |  |  |  |  |
